# CKS1 inhibition reveals vulnerabilities in leukemic stem cells with concomitant protection of healthy hematopoietic stem cells

**DOI:** 10.1126/scitranslmed.abn3248

**Published:** 2022-06-22

**Authors:** William Grey, Ana Rio-Machin, Pedro Casado-Izquierdo, Eva Grönroos, Sara Ali, Juho J. Miettinen, Findley Bewicke-Copley, Alun Parsons, Caroline A. Heckman, Charles Swanton, Pedro Cutillas, John Gribben, Jude Fitzgibbon, Dominique Bonnet

**Affiliations:** 1 Haematopoietic Stem Cell Laboratory, The Francis Crick Institute, London, U.K; 2 Centre for Genomics and Computational Biology, Bart’s Cancer Institute, London, U.K; 3 Cell signalling and proteomics group, Centre for Genomics and Computational Biology, Barts Cancer Institute, London, U.K; 4 Cancer evolution and genome instability laboratory, The Francis Crick Institute, London, U.K; 5 Institute for Molecular Medicine Finland – FINN, HiLIFE – Helsinki Institute of Life Science, iCAN Digital Precision Cancer Medicine Flagship, University of Helsinki, Helsinki, Finland; 6 Centre for Haemato-Oncology, Bart’s Cancer Institute, London, U.K.

**Keywords:** CKS1, Proteostasis, AML, Hematopoiesis, Chemotherapy

## Abstract

Acute myeloid leukemia (AML) is an aggressive hematological disorder comprising a hierarchy of quiescent leukemic stem cells (LSCs) and proliferating blasts with limited self-renewal ability. AML has a dismal prognosis, with extremely low two-year survival rates in the poorest cytogenetic risk patients, primarily due to the failure of intensive chemotherapy protocols to deplete LSCs, and the significant toxicity towards healthy hematopoietic cells. Whilst much work has been done to identify genetic and epigenetic vulnerabilities in AML LSCs, little is known about protein homeostasis in drug resistance and relapse. By targeting the proteostatic regulator CKS1, we demonstrate a dual role for CKS1-dependent protein degradation in reducing AML blasts *in vivo*, and importantly depleting LSCs, whilst inhibition of CKS1 has the opposite effect on normal hematopoiesis, protecting normal hematopoietic stem cells from chemotherapeutic toxicity. Together these findings demonstrate CKS1-dependent proteostasis is a key vulnerability in malignant stem cell biology.

## Graphical abstract

**Graphical Abstract:**
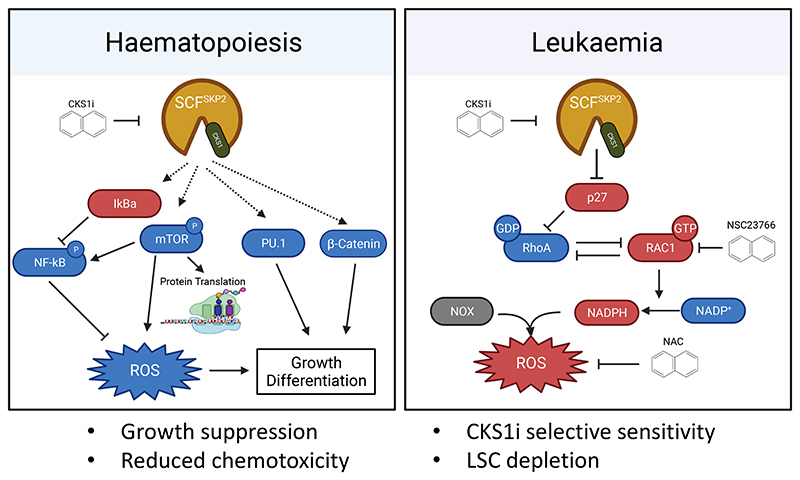

## Introduction

Acute myeloid leukemia (AML) is a heterogeneous, aggressive disease of the hematopoietic system, arising from hematopoietic stem/progenitor cells. The average two-year survival rate is 5-15% in poor risk, older AML patients (>65yr), demonstrating an unmet critical need for new therapeutic approaches(1). Fundamentally, leukemic stem cells (LSCs), the cancer stem cells (CSCs) of the hematopoietic system, are the origins of relapse in AML(2) and show significant plasticity from *de novo* disease through to relapse(3). Therefore, new approaches targeting AML LSCs are critical for improving AML prognosis. Recent developments, targeting the anti-apoptotic protein BCL2 (e.g. Venetoclax), has demonstrated that therapies affecting protein networks holds great promise for a wide variety of cancers, including the poorest prognosis AMLs(4, 5). Yet resistance still emerges through LSC adaptations(6, 7).

The key aim of CSC-targeted therapy is to selectively reduce CSCs without negatively affecting normal stem cells. Improved understanding of the biological differences between normal and malignant stem cells is needed to achieve selective CSC targeting, without toxicity to normal stem cells.

We previously reported a regulatory axis between the cyclin-dependent kinase subunits Cks1 and Cks2, and the mixed lineage leukemia 1 protein (Mll1), a key protein hijacked during neoplastic transformation of the hematopoietic system(8), and important for regulation of normal and cancer stem cells from multiple different tissues(9, 10). Cks1 and Cks2 have multifaceted overlapping and independent roles in balancing protein homeostasis (proteostasis) throughout the cell cycle, ensuring correct G0/G1 transition(11), chromatin separation(12–14) and DNA repair(11, 15, 16). Cks1 and Cks2 also possess CDK-independent functions, in concert with the SCF^SKP2^ and APC^CDC20^ E3 ubiquitin ligase complexes, important for selective protein degradation(11, 12, 17).

The ubiquitin proteosome system (UPS) is a highly regulated system that controls protein degradation and is essential for correct cellular protein homeostasis. It has been reported that up to 80% of cellular proteins are degraded by the UPS, demonstrating its importance in proliferation, survival, differentiation and drug resistance (18–21). Targeting the UPS has proved elusive in hematopoietic disorders. Broad spectrum inhibitors of protein degradation (e.g. Bortezomib) have shown increased by-toxicity without improvement of overall survival (22). Targeting less broad cullin-dependent protein degradation (e.g. Pevonedistat) was initially promising (23, 24), but trials have failed to significantly improve overall survival(25; NCT03268954). We previously demonstrated that pan-cullin inhibition can lead to cell cycle arrest in AML, whereas more specific inhibition of protein degradation targeting CKS1 leads to cell death (8). Indeed, small molecule inhibitors targeting SCF-SKP2-CKS1 are able to stabilise p27 protein levels and block cancer cells in G2/M phase of the cell cycle, leading to cell death, rather than cell cycle arrest (8, 26, 27).

In the current study, we have investigated the sensitivity of poor risk AML – a sub-classification with few treatment options – to protein phosphorylation and degradation inhibitors to reveal CKS1-dependent vulnerabilities. We demonstrate high efficacy of inhibiting CKS1-dependent protein degradation in reducing the LSC pool either as a single treatment or in combination with standard chemotherapy. In contrast, CKS1 inhibition has the opposite effect on normal hematopoiesis, improving stem cell functionality and conferring protection from chemotherapeutic toxicity. Together, these findings offer a new treatment paradigm for eradicating drug resistant LSCs whilst preserving healthy hematopoiesis.

## Results

### High expression of *CKS1B* dictates sensitivity of bulk AML to inhibition of CKS1-dependent protein degradation

The overexpression of *CKS1B* correlates with poor prognosis in a variety of solid tumors(28–30), but is an indeterminant factor in AML (Supp. Fig. 1A-D) despite a broad range of expression in normal and malignant hematopoiesis across multiple cohorts and datasets (Supp. Fig. 1E). *CKS1B* expression varies significantly between both normal and malignant hematopoiesis and within different hematopoietic subtypes (Kruksal-Wallis, p<2.2^-16^, Supp. Table 1), with intermediate expression in healthy hematopoietic stem cells (HSCs), and a broad range of expression in most AML cytogenetic subtypes compared to one of its key upstream proteostatic regulation partners *SKP2* (Supp. Fig. 1F).

**Figure 1:**
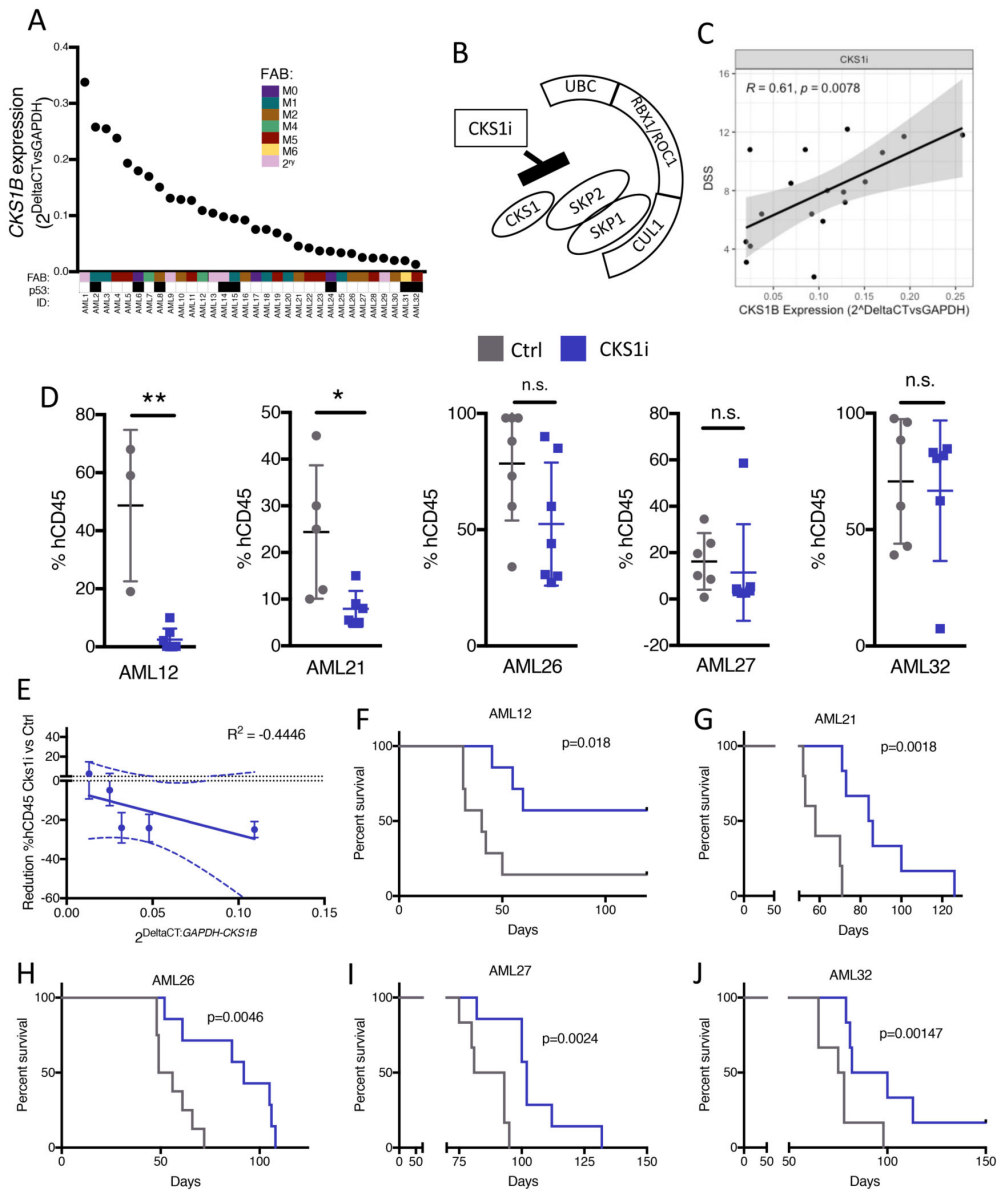
Inhibition of CKS1-dependent protein degradation kills AML blast. **A.** Expression of *CKS1B* (relative to *GAPDH*) in a poor risk AML cohort. FAB and p53 status are indicated for each patient (FAB color coded, p53 status: white = WT; black = mutant; N=32). **B.** Diagram of action for CKS1i binding and inhibition of the SCF^SKP2-CKS1^ ubiquitin ligase complex. **C.** Correlation between CKS1i drug sensitivity (DSS) and *CKS1B* expression (relative to *GAPDH*) **D.** Percentage of human CD45^+^ cells of total CD45^+^ cells in mouse bone marrow aspirations one week after chemotherapy (week 6). **E.** Correlation between *CKS1B* expression and reduction in human AML burden post CKS1i treatment. **F-J.** Kaplan Meier plots and p value calculated (Mantel-Cox test) for each individual PDX control and CKS1i treated cohort. Each data point represents one mouse. A Student’s *t*-test was used to calculate significance of difference for all graphs unless otherwise stated. * p<0.05; **p<0.005.

We hypothesized that high *CKS1B* expression in AML may provide a selective susceptibility to inhibition of either CDK-CKS1-dependent phosphorylation or SCF-CKS1-dependent protein degradation (SCF^SKP2-CKS1^ E3 ligase inhibitor, hereafter referred to as CKS1i)(26, 27). To address this key question, we screened a cohort of cytogenetically poor risk AMLs, spanning a variety of morphological (FAB) and molecular subtypes, with a broad range of *CKS1B* expression (Figure 1A, Supp. Table 2) for sensitivity to a range of CDK inhibitors, a broad-spectrum protein degradation inhibitor (Bortezomib), and specific inhibitors of the SCF^SKP2-CKS1^ E3 ubiquitin ligase complex (Pevonedistat and CKS1i; Figure 1A-B, Supp. Fig. 2A-B, Supp. Table 3).

Whilst CDK inhibition resulted in fewer than 50% of patients demonstrating robust drug sensitivity (DSS), protein degradation inhibitors demonstrated increased drug sensitivity of AML blasts grown *in vitro* as the spectrum of inhibition increased (Supp. Fig. 2A). Since failure of broad-spectrum protein degradation inhibitors has been reported previously, and we reported induction of quiescence rather than cell death by Pevonedistat(8), we investigated whether inhibition of more specific CKS1-dependent protein degradation could be more effective. Indeed, knockdown of *CKS1B* in AML results in dose- and time-dependent reduction in viability (Supp. Fig. 2C-E), and CKS1i drug sensitivity directly correlates with *CKS1B* expression in poor risk AML patients (R=0.61, p=0.0078; Figure 1C), with clear separation of high and low DSS (Supp. Fig 2F). Separating patients at the 50^th^ percentile by *CKS1B* expression revealed significantly increased drug sensitivity in *CKS1B^high^* versus *CKS1B^low^* AML patients (Supp. Fig 2F), indicating that RNA expression of *CKS1B* could be a selection criterion for targeting SCF^SKP2-CKS1^ dependent protein degradation in AML. Additional characterization of patient phenotypes indicated that white blood counts at diagnosis are similar between *CKS1B^high^* and *CKS1B^low^* patients and CKS1i responders and non-responders, and both groupings covered an array of mutational profiles, with a multivariate analysis demonstrating only *CKS1B* expression correlates with CKS1i sensitivity (Supp. Fig. 2G-I, Supp. Tables 4, 5 & 6).

In order to investigate the effect of CKS1i on primary patient AML *in vivo*, we selected five primary patient samples with a range of *CKS1B* expression to engraft in immunodeficient NSG mice (Supp. Table 2). A single course of CKS1i (10mg/kg, 5 days I.P.) significantly reduced the leukemic burden in mice engrafted with patient AMLs carrying the highest *CKS1B* expression (AML12 and AML21). A trend towards reduced AML burden was seen at an intermediate level of *CKS1B* expression (AML26), but CKS1 inhibition had no significant effect on bulk AML for patients with the lowest *CKS1B* expression (AML27 and AML32; Figure 1D). As such, *CKS1B* expression levels directly correlated with acute tumor reduction *in vivo* (R=-0.446; Figure 1E). Interestingly, all CKS1i treated AML xenografts showed a delay in AML bone marrow colonisation over time, regardless of tumor reduction immediately post-CKS1i treatment (Supp. Fig. 3) and significantly improved overall survival compared to untreated controls (Figure 1F-J), indicating that CKS1i treatment had additional effects beyond acutely reducing bulk leukemic burden of *CKS1B^high^* AML.

### CKS1-dependent degradation is a specific vulnerability in Leukemic Stem Cells

Whilst reducing leukemic blast count is the current backbone of clinical chemotherapeutic protocols and required to release leukemic cell-mediated suppression of normal hematopoietic cells, these approaches do not target quiescent LSCs, the subset of cells at the origin of relapse *in vivo*(31). The observed effect on bone marrow colonisation and overall survival upon CKS1i treatment in *CKS1B*^low^ AMLs could indicate a specific mechanism of action of CKS1i on LSCs. Indeed, LSCs are rare and bulk *CKS1B* expression does not account for LSC-specific *CKS1B* dependency.

**Figure 2:**
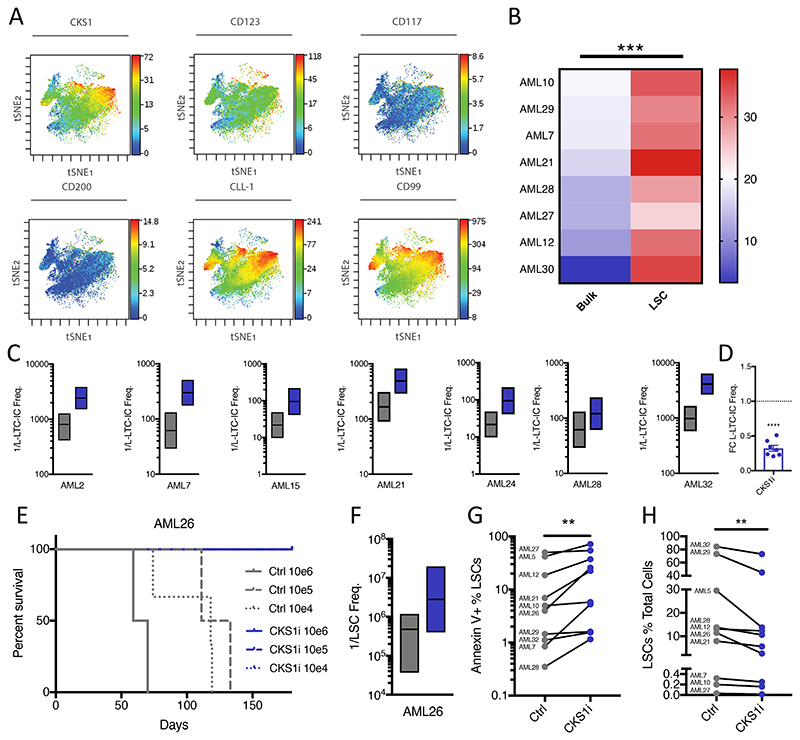
AML LSCs have high CKS1 and are critically sensitive to CKS1i. **A.** *t*-stochastic neighbor embedding of patient AML7 illustrating co-expression of CKS1 protein with key LSC cell surface markers. **B.** Median intensity of CKS1 protein levels in bulk AML versus LSCs. **C.** Individual 1/L-LTC-IC frequencies with upper and lower limits for each patient tested. Control (Grey) vs CKS1i (Blue). **D.** Fold change L-LTC-IC frequency, CKS1i treatment versus control for all patient samples tested. **E.** Overall survival of AML26 secondary transplantation with the indicated cell doses from primary treatment mice. **F.** Estimated LSC frequency of secondary transplanted AML26. Control calculated at week 6, CKS1i calculated at the end point of the experiment. **G.** Percentage of apoptotic (Annexin V positive) LSCs in control and CKS1i treated primary patient AML *in vitro* 24 hours after treatment. **H.** Percentage of LSCs in total AML cells in control and CKS1i treated primary patient AML *in vitro* 24 hours after treatment. A Student’s *t*-test was used to calculate significance of difference for all graphs unless otherwise stated. * p<0.05; **p<0.005; *** p< 0.0005.

Transcriptomic analysis of patient AMLs at single cell resolution revealed subsets of AML expressing *CKS1B* clustering with LSC genes (Supp. Fig 4A-B). To better quantify LSC-dependency on CKS1 in primary patient AML, we investigated CKS1 protein levels at single cell resolution. Mass cytometry-based *t*-stochastic neighbor embedding demonstrated strong association of CKS1 protein levels with a range of immunophenotypic and functional LSC markers (Figure 2A, Supp. Fig. 4C). When focussing on primary patient immunophenotypic LSC subpopulations (CD200^+^CD99^+^CLL-1^+^CD123^+^CD117^+^, Supp. Fig. 5A), CKS1 protein levels are significantly higher than bulk AML (Figure 2B). Similarly, immunophenotypic LSCs have increased levels of proteins important for both stem cell functionality and drug resistance (e.g. BCL2, active β-catenin; Supp. Fig. 5B).

To assess the functional effect of CKS1i on LSCs we used the leukemic-long-term culture initiating cell assay (L-LTC-IC). All patients showed significant reduction in L-LTC-IC frequency, demonstrating a direct effect of CKS1i treatment on LSC functionality (Figure 2C-D, Supp. Fig. 5C). In addition, primary human AML cells recovered from AML26 xenografts were secondarily transplanted in limiting dilution. No xenografts carrying CKS1i treated AMLs showed overt signs of ill-health, whereas control xenografts died within 150 days (Figure 2E). Analysis of human bone marrow engraftment of secondary xenograft mice revealed a significant reduction in LSC frequency by CKS1i treatment (Figure 2F, Supp. Fig. 5D). In agreement, when cultured *in vitro*, patient AMLs treated with CKS1i show increased apoptosis in the LSC compartment (Figure 2G) and a reduction in LSCs compared to total AML blasts (Figure 2H).

These data demonstrate that LSCs have high levels of CKS1 and CKS1i is efficient at targeting the LSC compartment. The reduction of LSCs by CKS1i indicates a clear route to combating AML in all patients independent of bulk *CKS1B* expression.

### CKS1 inhibition protects healthy hematopoiesis from chemotherapeutic toxicity

**Figure 3:**
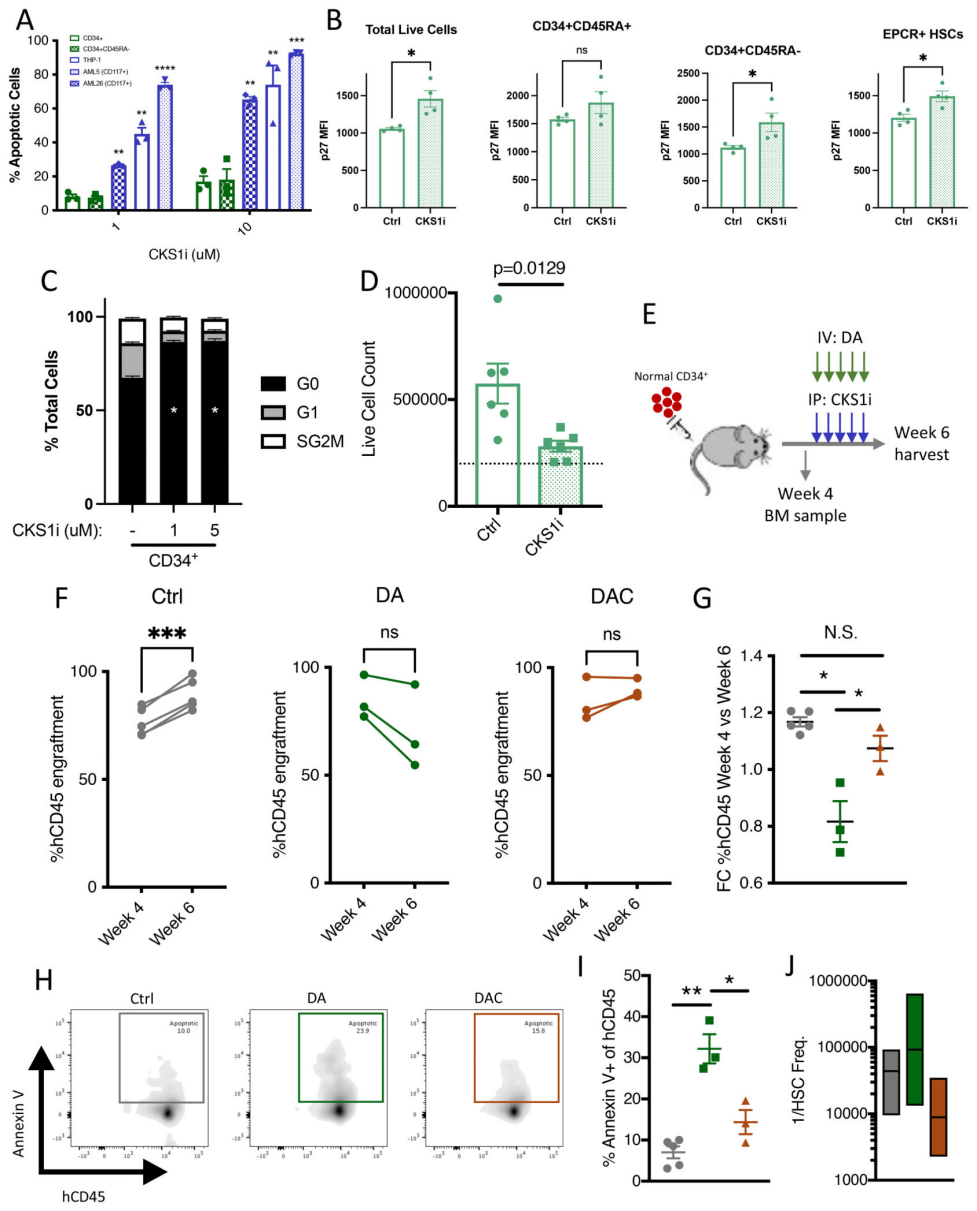
CKS1i protects normal hematopoietic cells from chemotherapeutic toxicity by suppressing the cell cycle. **A.** Percentage Annexin V positive apoptotic cells for the indicated cell types in response to increasing concentrations of CKS1i. **B.** p27 protein mean fluorescent intensity measured in CD34^+^ cells cultured with CKS1i (1μM) in the indicated cell populations. **C.** Cell cycle profile and **D.** Total cell count of CD34^+^ cells treated with the indicated doses of CKS1i (1μM for live cell count) for 24 hours. **E.** Illustration of CD34^+^ engraftment and chemotherapeutic treatment in NSG mice. **F.** Change in percentage human CD45^+^ of total CD45 at the indicated time points for Control (Ctrl), Doxorubicin/Cytarabine (DA) and Doxorubicin/Cytarabine plus CKS1i (DAC) treatments. **G.** Fold change of the percentage of human CD45 cells at week 4 and 6 for the indicated treatments (Control = Grey, DA = Green, DAC = Brown). **H.** Representative flow plots and **I.** Percentage of total cells annexin V positive after 6 weeks *in vivo* for human CD45 cells with the indicated treatment conditions (Ctrl N=5, DA N=3, DAC N=3). **J.** HSC frequency calculated by limiting dilution secondary transplantation of human CD45^+^ cells retrieved from primary mice (Control = Grey, DA = Green, DAC = Brown). A Student’s *t*-test was used to calculate significance of difference unless otherwise stated. * p<0.05; **p<0.005.

Contrary to primary patient AML LSCs and AML cell lines, healthy umbilical cord blood derived CD34^+^ and the more primitive CD34^+^CD45RA^-^ compartment do not undergo apoptosis in response to CKS1i (Figure 3A). Where AML cells accumulate in S-G2-M phases of the cell cycle (Supp. Fig. 5E), healthy CD34^+^ cells increase p27 levels in primitive fractions (Figure 3B) and become significantly more quiescent (Figure 3C), leading to fewer cells in culture over time (Figure 3D).

By inducing quiescence and limiting cell growth, CKS1i would reduce the ability to incorporate nucleotide analogues (e.g. Cytarabine) and the toxicity of topoisomerase inhibitors (e.g. Doxorubicin). We hypothesized that this could place CKS1i as a “chemoprotective agent” during classical induction chemotherapy in AML, protecting healthy hematopoietic cells from chemotherapeutic killing.

To investigate this hypothesis, we engrafted healthy umbilical cord blood derived CD34^+^ cells in NSG mice and treated the mice with the clinical chemotherapy protocol of cytarabine plus doxorubicin (5+3 days)(33), in the presence or absence of CKS1i (Figure 3E). Human bone marrow engraftment increased in untreated control mice between weeks 4 and 6 as would be expected. Treatment at week 4 with doxorubicin/cytarabine (DA) reduced bone marrow engraftment by week 6, significantly reducing the expansion of human cells compared to control, but addition of CKS1i (DAC) was able to rescue this effect, returning expansion of human cells to comparable levels to control (Figure 3F-G). Better engraftment at week 6 was complemented by a reduction in apoptotic human cells in the bone marrow of recipient mice (Figure 3H-I), indicating that CKS1i treatment prevents DA-induced cell death in normal hematopoietic cells. Secondary transplantation of human cells obtained from primary treatment mice revealed a significant increase in HSC frequency after CKS1i treatment, rescuing DA effects on HSCs (Figure 3J). Indicating that CKS1i protects healthy HSCs from chemotherapy induced depletion.

Outside of the hematopoietic system a key side-effect of induction chemotherapy for AML is severe gut by-toxicity, often resulting in intestinal dysfunction and infection(34, 35). In agreement with the effects on normal HSPCs, DA treatment induced increased proliferation of intestinal crypts (Supp. Fig. 6A-B) and resulted in fewer LGR5^+^ crypts post-chemotherapy (Supp. Fig. 6C-D). Both phenotypes were rescued by the addition of CKS1i, returning to comparable levels to untreated controls.

These data demonstrate that CKS1i has the opposite effect on healthy tissue compared to AML, and suppression of growth induced by CKS1i can be chemoprotective for healthy tissue during clinically used chemotherapy.

### Divergent cellular responses to CKS1i by healthy and malignant hematopoietic cells

**Figure 4:**
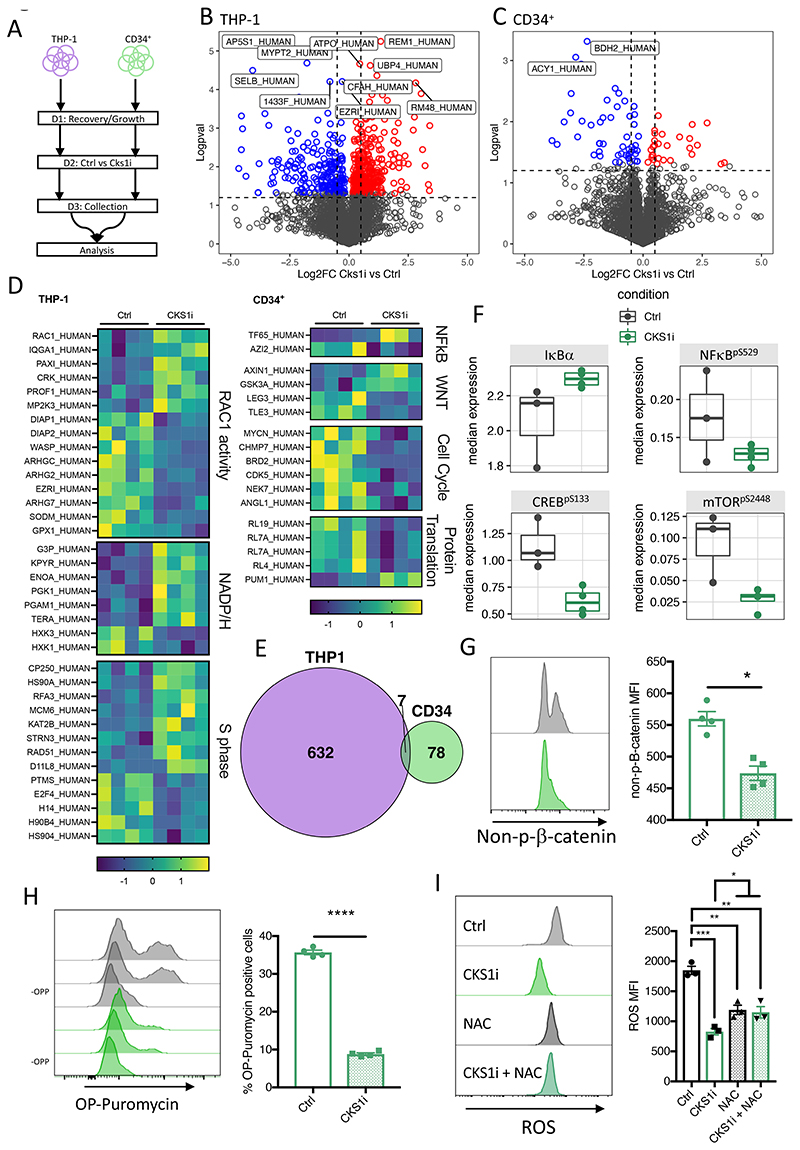
CKS1i treatment induces divergent proteomic alterations in normal and malignant hematopoietic cells. **A.** Workflow for timescale of cell preparation for mass spectrometry analysis. Volcano plots for proteomic alterations in **B.** THP-1 and **C.** CD34^+^ cells in response to CKS1i (1μM). **D.** Key differentially abundant proteins in THP-1 or CD34^+^ cells in response to CKS1i (N=4 per condition). **E.** Venn diagram depicting overlap of differentially expressed proteins between THP-1 and CD34^+^ cells. **F.** Median expression of key intracellular signalling markers identified in CyTOF analyses after CKS1i treatment (Ctrl N=3, CKS1i N=4). **G.** Representative flow plots and quantified mean fluorescence intensity for non-phosphorylated β-catenin in CD34^+^ cells grown for 48 hours in control conditions or treated with CKS1i (N=4). **H.** Representative flow plots (including cells grown without OP-Puromycin; -OPP) and % total OP-Puromycin incorporation in CD34^+^ cells grown for 48 hours in control conditions or treated with CKS1i. OP-Puromycin was added 1hr prior to collection and fixation of cells (N=4). **I.** Representative flow plots and quantified mean fluorescence intensity of intracellular reactive oxygen species (ROS) in CD34^+^ cells grown for 48 hours in control conditions or treated with CKS1i (1μM) or NAC (1.25mM; N=3 per condition). * p<0.05; **p<0.005; ***p<0.0005; ****p<0.0001.

To investigate the mechanism by which CKS1i induces divergent responses between healthy and malignant hematopoietic cells, we carried out proteomic analysis of *CKS1B^high^* AML cell lines, which demonstrate direct correlation between *CKS1B* expression and CKS1i response, phenocopying primary patient AML (Supp. Fig. 7, Supp. Table 6), and umbilical cord blood derived healthy CD34^+^ HSPCs, with and without CKS1i treatment *in vitro* (1μM; Figure 4A).

CKS1i treatment induced ~7.5x more significantly altered proteins in THP-1 cells compared to healthy CD34^+^ (Figure 4B-C). Differentially abundant cell cycle proteins demonstrate the divergent responses to CKS1i by healthy and malignant hematopoietic cells. Indeed, downregulation of cell cycle drivers and protein translation machinery in CD34^+^ cells and upregulation of S phase promoting proteins in AML cells, with relatively few overlapping proteins (<10%), explains divergent cell cycle responses (Figure 4D-E).

Furthermore, key proteins differentially abundant in CD34^+^ cells and not AML were integrated in three pathways fundamental to normal hematopoiesis: Wnt signalling, cell cycle control and NFkB signalling (Figure 4D, Supp. Fig. 8A). To investigate the changes in these key signalling pathways at single cell resolution we carried out mass cytometry with a panel of cell surface and intracellular markers covering signalling pathways important for HSPC proliferation, differentiation and stem cell self-renewal (36).

Pseudo-bulk-level multidimensional scaling demonstrated a convergence of individual CD34^+^ donors upon treatment with CKS1i (Supp. Fig. 8B). These differences in CD34^+^ cells after CKS1i treatment were largely due to a reduction in abundance of intracellular signalling markers (Supp. Fig. 8C), particularly IκBα/NFκB signalling, CREB and mTOR phosphorylation (Figure 4F, Supp. Fig. 8D) and reduced proliferating cells (Figure 3C). Changes that were not observed in bulk AML or AML LSCs in response to CKS1i (Supp. Fig. 8E). In addition, the protein levels of differentiation regulators such as PU.1 were also reduced (Supp. Fig. 8D), indicating a potential block in differentiation. Fewer cells had active non-phosphorylated β-catenin, demonstrating that the Wnt pathway – a fundamental pathway requiring a tight balance for normal hematopoiesis to proceed – was suppressed (Figure 4G, Supp. Fig. 8D).

Reduction of metabolically active markers (e.g. mTOR^pS2448^), inflammatory responses (e.g. NFkB^pS529^), and suppression of the translation machinery in our mass spectrometry analyses resulted in reduction of protein translation in CKS1i treated CD34^+^ cells (Figure 4H). Together, these signalling pathways are fundamental to the control of stress responses and particularly important to prevent the accumulation of lethal ROS in HSCs(37). In agreement, CKS1i treatment reduced intracellular ROS in CD34^+^ cells (Figure 4I). Interestingly CKS1i-dependent reduction of ROS surpassed that of NAC treatment, with no additive effects of CKS1i and NAC (Figure 4I). This leads to improved stem cell frequency of CD34^+^ cells cultured in the presence of CKS1i (Supp. Fig. 8F).

The substantial changes in these key pathways are hallmarks of suppression of growth and differentiation, rather than an induction of cell death by CKS1i, confirming our functional data (improved HSC frequency when treated with CKS1i alone and protecting HSCs from the toxicity of Cytarabine/Doxorubicin; Figure 3I, Supp. Fig. 8F).

### CKS1i induces an integrated molecular switch in AML cells driving RAC1 activity and NADP/H metabolism

**Figure 5:**
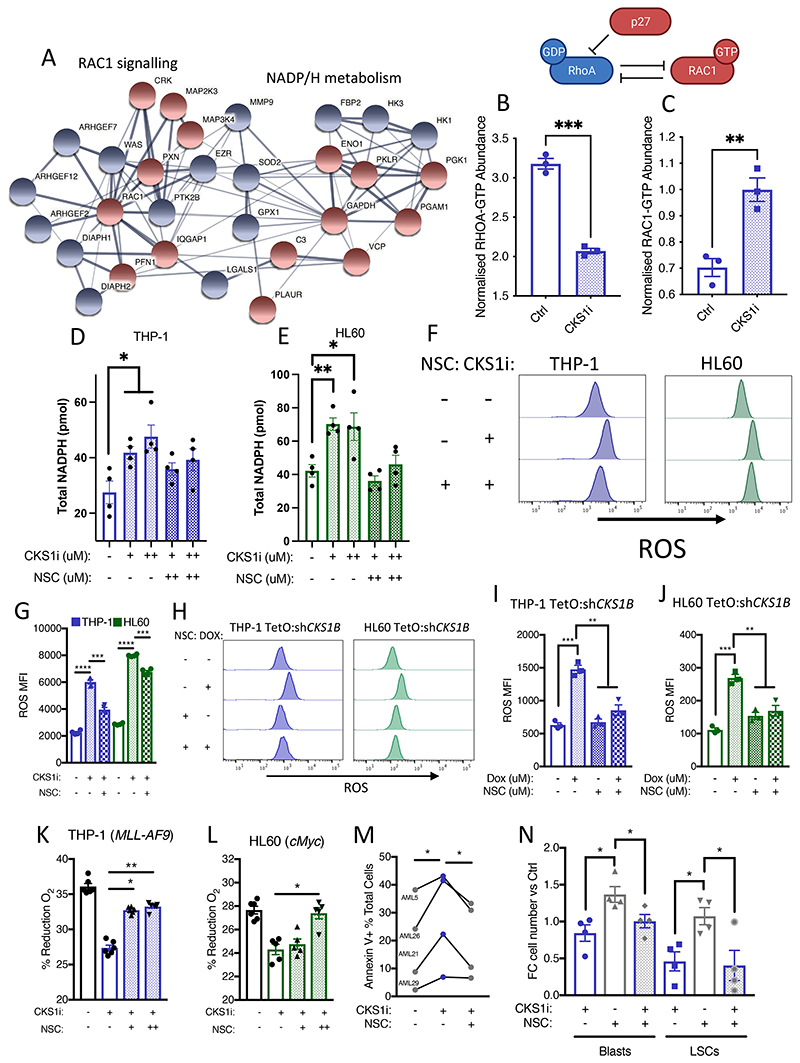
The SCF^SKP2-CKS1^ complex controls RAC1/NADPH/ROS signalling. **A.** String network analysis of key differentially abundant proteins in THP-1 cells treated with CKS1i. Red indicates upregulated, and blue indicates downregulated in response to CKS1i treatment. **B.** RHOA-GTP and **C.** RAC1-GTP abundance in THP-1 cells control or treated with CKS1i (1 μM) for 24 hours (N=3 independent experiments). Total NADPH (pmol) in **D.** THP-1 and **E.** HL60 cells treated with the indicated doses of CKS1i (+ = 1μM, ++ = 5μM) or NSC23766 (NSC; + = 0.1μM, ++ = 1μM) for 8 hours (N=4 independent experiments per cell line and treatment). **F.** Representative flow plots and **G.** Quantified mean fluorescence intensity of intracellular reactive oxygen species (ROS) in the indicated cell lines in response to CKS1i (+ = 1μM) and NSC (+ = 0.1μM) treatment (N=3 per cell line and treatment). **H.** Representative flow plots and **I-J.** Quantified mean fluorescence intensity of intracellular reactive oxygen species (ROS) in the indicated cell lines in response to *CKS1B* knockdown and NSC (+ = 0.1μM) treatment (N=3 per cell line and treatment). **K-L.** Viability represented by percentage reduction O_2_ of the indicated cell lines in response to the indicated concentrations of CKS1i and NSC23766 (N=5 per cell line and treatment, except THP-1 where N=6), CKS1i (+ = 1μM) and NSC (+ = 0.1μM, ++ = 1μM). **M.** Percentage Annexin V positive apoptotic primary patient AML samples treated with the indicated doses of CKS1i (+ = 1μM) and NSC (+ = 0.1μM). **N.** Fold change cell number versus control for total AML (Blasts) and LSCs with the indicated treatments (CKS1i + = 1μM and NSC + = 0.1μM) 24 hours after treatment *in vitro*. A Student’s *t*-test was used to calculate significance of difference for all graphs. * p<0.05; **p<0.005; *** p< 0.0005; **** p< 0.0001.

Proteomic alterations mediated by CKS1i in AML revealed key changes beyond S phase accumulation, with modulators of RAC1 and NADP/H activity significantly altered (Figure 5A, Supp. Fig. 9A-B).

Total RAC1 protein abundance was increased (Supp. Fig. 9C), as well as key interactors, such as Paxillin and CRK, after CKS1i treatment (Figure 5A). Mechanistically, inhibition of the SCF^SKP2-CKS1^ complex leads to accumulation of p27 (Supp. Fig. 9D), which inhibits RHOA activity (Figure 5B, Supp. Fig. 9E) (38). This reduces the activity of RAC-GAPs, to maintain RAC1 in its GTP bound state (39), working in concert with RAC1 signalling pathway members to significantly increase the amount of RAC1-GTP in AML after CKS1i treatment (Figure 5C, Supp. Fig. 9F). RAC1-GTP together with NOXA(p67^Phox^) regulates NADP to NADPH conversion – providing a pool for NADPH oxidases to produce ROS(40) – and CKS1i significantly altered a range of NADP/H metabolic regulators (Figure 5A). Thus, we evaluated the abundance and ratio of NADP/NADPH upon CKS1i treatment.

CKS1i induced a dose dependent increase of NADPH in AML cells (Figure 5D-E, Supp. Fig. 10A-D). The accumulation of NADPH is dependent on RAC1-GTP activity, as CKS1i induction of NADPH was rescued by the RAC1 inhibitor NSC23766 (NSC, Figure 5D-E. Supp. Fig. 10A-D).

Sensitivity of the RHOA-RAC1 axis to CKS1i correlates with p27 stabilization (Supp. Fig. 9D) and IC50 values in *CKS1B^high^* and *CKS1B^low^* AML cell lines (Supp. Fig. 7), further demonstrating the dose-dependent sensitivity to CKS1i based on *CKS1B* expression.

Together, these data demonstrate that inhibition of the SCF^SKP2-CKS1^ complex induces an integrated molecular switch, with regulation of RAC1/NADPH activity maintained by convergent signalling pathways.

### Inhibition of SCF-SKP2-CKS1 drives lethal ROS accumulation in AML

CKS1i-induced RAC1 activity and NADPH accumulation led to significantly increased intracellular ROS in AML (Figure 5F-G, Supp. Fig. 10E-F), a phenotype conserved upon *CKS1B* knockdown (Figure 5H-J), indicating that CKS1 is critical to balance ROS abundance.

Inhibition of RAC1 rescued intracellular ROS accumulation induced by CKS1i or *CKS1B* knockdown (Figure 5F-J, Supp. Fig. 10 E-F), and at higher doses is able to rescue CKS1i induced reduction in cell viability (Figure 5K-L, Supp. Fig. 10G-H).

Primary AML grown *in vitro* demonstrates similar sensitivity to CKS1i treatment, with induction of apoptosis in both bulk AML (Figure 5M) and importantly the LSC fraction (Figure 5N). However, whereas RAC1 inhibition can improve the growth of AML, CKS1i effects on LSCs are dominant, maintaining LSC depletion during double treatment (Figure 5N, Supp. Fig 10I-K).

**Figure 6:**
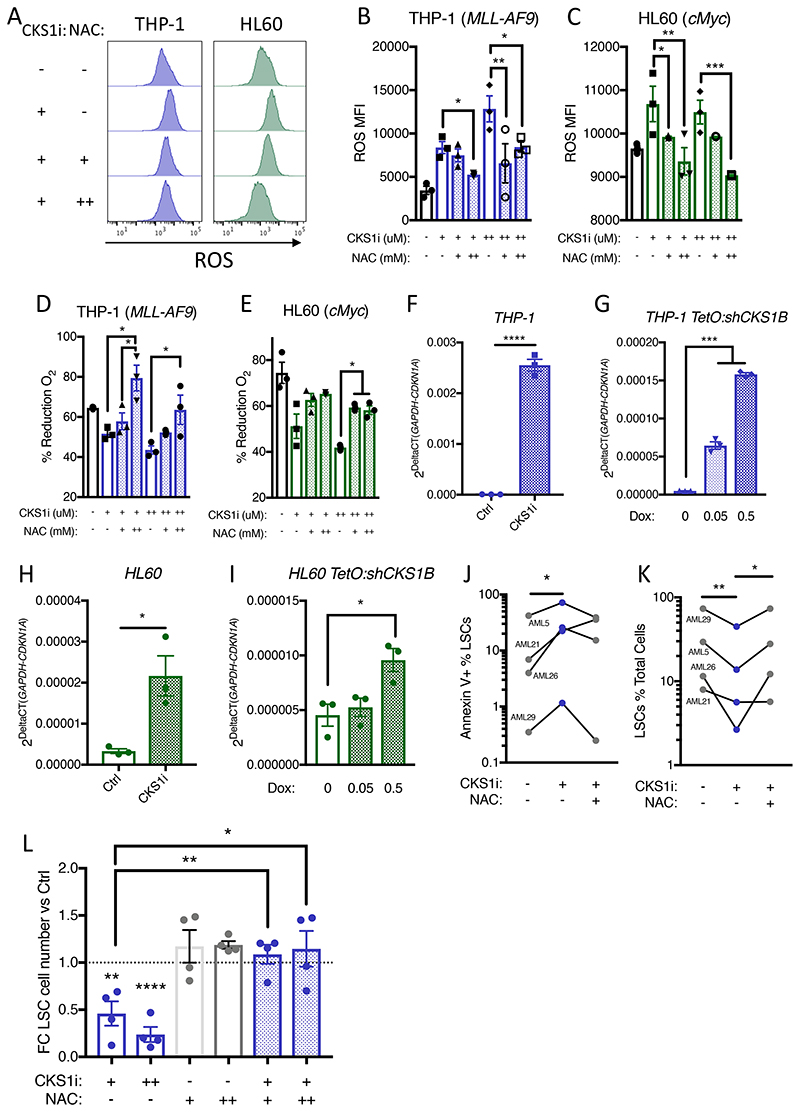
CKS1i treatment depletes LSCs by inducing lethal ROS. **A.** Representative flow plots and **B-C.** Quantified mean fluorescence intensity of intracellular reactive oxygen species (ROS) in the indicated cell lines in response to CKS1i (+ = 1μM, ++ = 5μM) and NAC (+ = 1.25mM, ++ = 2.5mM) treatment (N=3 per cell line and treatment). **D-E.** Viability represented by percentage reduction O_2_ of the indicated cell lines in response to the indicated concentrations of CKS1i (+ = 1μM, ++ = 5μM) and NAC (+ = 1.25mM, ++ = 2.5mM; N=3 per cell line). Quantitative PCR analysis of *CDKN1A* expression in **F.** THP-1 cells treated with CKS1i, **G.** THP-1 cells with *CKS1B* knockdown, **H.** HL-60 cells treated with CKS1i and **I.** HL60 cells with *CKS1B* knockdown for 24 hours (N=3). **J.** Induction of apoptosis (Annexin V+) in primary patient LSCs in response to CKS1i and NAC (CKS1i + = 1μM, NAC + = 1.25mM) 24 hours after treatment *in vitro*. **K.** Percentage LSCs of total primary patient AML blasts in response to CKS1i and NAC (CKS1i + = 1μM, NAC + = 1.25mM) 24 hours after treatment *in vitro*. **L.** Fold change absolute number of primary patient LSCs in the indicated treatments versus control (CKS1i + = 1μM, CKS1i ++ = 5μM, NAC + = 1.25mM, NAC ++ = 2.5mM) 24 hours after treatment *in vitro*. A Student’s *t*-test was used to calculate significance of difference for all graphs. * p<0.05; **p<0.005; *** p< 0.0005; **** p<0.0001.

As the antioxidant N-acetyl-L-cysteine (NAC) is well known to scavenge intracellular ROS to reverse the negative effects of ROS on HSCs/LSCs, we tested whether NAC could reduce intracellular ROS accumulation and rescue survival. Indeed, NAC was able to reduce intracellular ROS in CKS1i treated AML (Figure 6A-C), and at higher doses NAC reversed CKS1i-dependent reduction in viability, demonstrating that CKS1i kills AML through accumulation of lethal levels of ROS (Figure 6D-E). Additionally, increased intracellular ROS by CKS1i, or knockdown of *CKS1B*, led to induction of *CDKN1A* expression (Figure 6F-I), a known downstream effect of ROS causing cell cycle arrest and apoptosis.

Patient LSCs must maintain low ROS for survival(41), and treatment of primary patient AML *in vitro* with CKS1i induced apoptosis in the LSC fraction and reduced both the proportion and total number of LSCs compared to control conditions (Figure 6J-L, Supp. Fig. 10 L-M). NAC treatment rescued CKS1i-induced LSC depletion in three out of four cases, returning LSC number to levels similar to control conditions (Figure 6J-L).

These data demonstrate that AML requires SCF^SKP2-CKS1^ functions to maintain a fine balance of intracellular ROS, which is critical for LSC maintenance *in vivo*. Ultimately, the significant increase in ROS, and the reduction in LSCs driven by CKS1i, indicates a clear pathway to target *CKS1B*^high^ LSCs *in vivo*, regardless of bulk *CKS1B* status in AML.

### Combining CKS1 inhibition with induction chemotherapy simultaneously reduces LSCs, protects normal HSCs and improves overall survival

**Figure 7:**
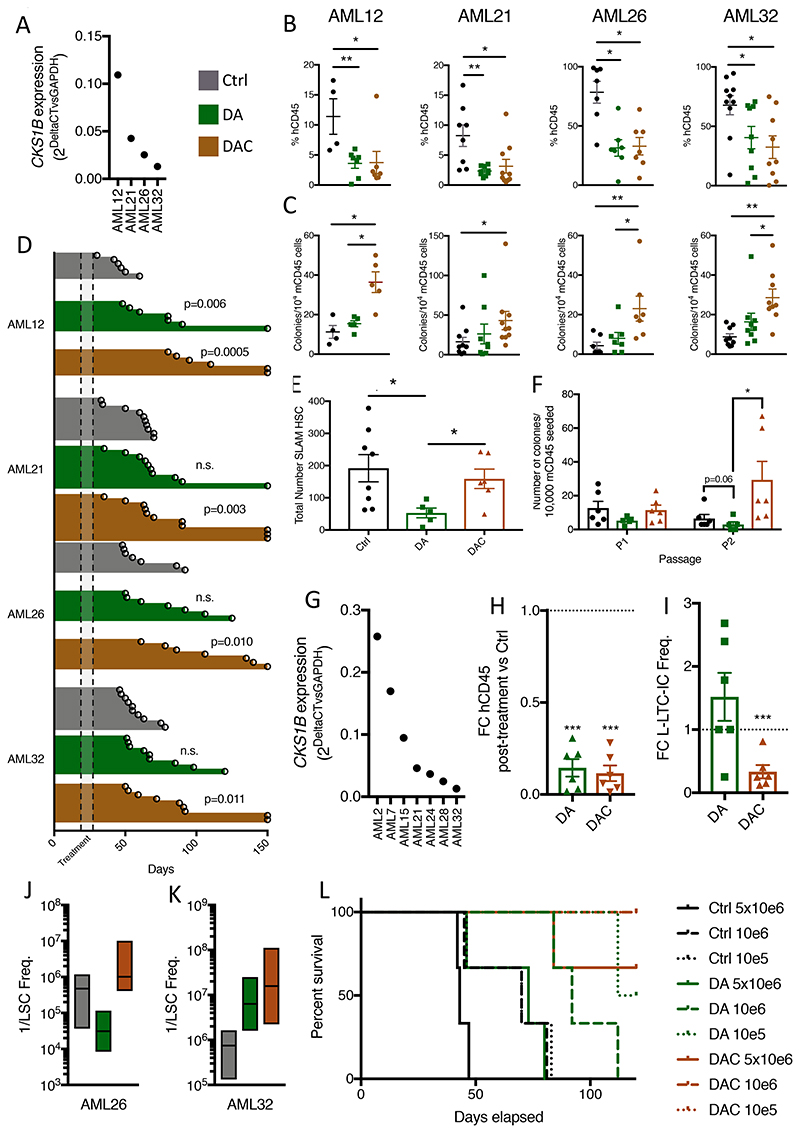
Combination of induction chemotherapy and CKS1i reduces AML burden and LSC potential whilst protecting resident hematopoietic cells. **A.** *CKS1B* expression (relative to *GAPDH*) for patient AMLs tested *in vivo*. **B.** Percentage of human CD45^+^ cells of total CD45^+^ cells in mouse bone marrow aspirations one week after chemotherapy (week 6). **C.** Colony forming units per 10,000 mouse CD45^+^ cells extracted from week 6 bone marrow aspirations. **D.** Swimmer plots and p values calculated (Mantel-Cox test) for each individual PDX Control and treated mouse cohort. Each data point represents one mouse and days survived are presented. Treatment interval is illustrated as annotated. **E.** Total number of murine Long-term HSCs obtained from bone marrow of mice at the final survival time point (Ctrl N=8, DA N=5, DAC N=5). **F.** Serial colony forming units per 10,000 mouse CD45^+^ cells obtained from BM of mice at the final survival time point (Ctrl N=6, DA N=5, DAC N=6). **G.** *CKS1B* expression (relative to *GAPDH)* for patient AMLs tested in L-LTC-IC. **H.** Fold change live human CD45^+^ cells, indicated treatments versus control, after two weeks of co-culture. **I.** Fold change of L-LTC-IC frequency of indicated treatment versus control, after 7 weeks of co-culture. **J-K.** LSC frequency in secondary transplanted mice injected with AML26 and AML32 at limiting dilution 6 weeks post-transplantation. **L.** Kaplan-Meier survival curve for AML32 secondary mice up to 120 days. A Student’s *t*-test was used to calculate significance of difference for all graphs unless otherwise stated. * p<0.05; **p<0.005; *** p< 0.0005.

To test the potential for combining classical DA chemotherapy with CKS1i (DAC) in AML, we transplanted NSG mice with primary AML samples of varying *CKS1B* expression (Figure 7A). After stratifying for engraftment at week 4, we treated the mice with either DA or DAC. One-week post chemotherapy, xenografts showed strong reduction in leukemic burden in both DA and DAC treatment cohorts for all AMLs, regardless of *CKS1B* expression (Figure 7B). At the same time point, resident murine CD45^+^ cells co-extracted from aspirated tibias had significantly higher colony forming potential upon the addition of CKS1i compared to untreated mice and DA treated mice (Figure 7C), indicating that CKS1i treatment can selectively reduce AML, whilst simultaneously protecting normal HSPCs colony forming potential. Overall, DA treatment was only able to significantly improve survival of one patient AML xenograft, due to the extensive by-toxicity combined with AML burden in NSG mice. Importantly, addition of CKS1i improved overall survival of all patient AML xenografts, with many xenograft mice surviving up to 150 days (Figure 7D, Supp. Fig. 11A-D).

Examination of the normal hematopoietic compartment of xenografted mice at the end point of survival revealed a severe reduction in total number of long-term HSCs (LT-HSCs) in the DA treated group, whereas addition of CKS1i to DA abolished this effect, rescuing LT-HSC number (Figure 7E). In addition, the serial colony forming ability of normal murine HSPCs was improved in DAC conditions, indicating that rescued HSPCs are functional (Figure 7F).

We and others have documented the refractory nature of LSCs to induction chemotherapy(42), and we set out to investigate the potential conflict or beneficial contribution between DA and CKS1i. In *ex vivo* conditions, both *CKS1B*^high & low^ AMLs (Figure 7G) showed significant reduction in total cell number one week after DA or DAC treatment (Figure 7H), yet whilst DA treatment enriched for L-LTC-IC frequency in three of the six patients, addition of CKS1i markedly reduced L-LTC-IC frequency in all patients compared to control and DA treated conditions (Figure 7I & Supp. Fig 11E-F).

Finally, to investigate the reduction in LSC frequency conferred by CKS1i *in vivo*, we engrafted AML cells obtained from AML26 and AML32 (which had the smallest improvement in overall survival after chemotherapy) in secondary recipients in limiting dilution. Whilst control AMLs retained strong LSC frequency and show robust engraftment after six weeks, this was increased by DA treatment in AML26 and was notably reduced in AML32 (Figure 7J-K, Supp. Fig. 12A-B). The addition of CKS1i counteracted the effect of DA by decreasing the LSC frequency in AML26 and further reducing LSC frequency in AML32 compared to DA and control mice, demonstrating strong reduction in LSCs after CKS1i treatment independent of the response to DA treatment (Figure 7J-K, Supp. Fig. 12A-B).

Overall secondary DA-AML mice survived significantly longer than controls, and DAC-AML treated mice showed further improvement in survival, with no overt signs of sickness at 150 days in six of seven cases for both AML26 and AML32 (Figure 7L, Supp. Fig. 12C).

Together, these data indicate that inhibition of CKS1-dependent protein degradation in combination with frontline chemotherapy is a more effective strategy to reduce the LSC pool, whilst protecting normal HSCs from chemotherapeutic toxicity.

## Discussion

The complex paradigm of selectively targeting CSCs whilst simultaneously preserving normal stem cells is a major challenge in cancer therapy, and the study of normal and malignant hematopoietic stem cells has played a major role in understanding CSC biology(43). In this study, we demonstrate that CKS1 is a key protein in this paradigm, with LSCs expressing higher CKS1 than most AML blasts, providing a selective vulnerability of LSCs to inhibition of the SCF^SKP2-CKS1^ E3 ubiquitin ligase complex, while sparing normal HSCs from chemotherapeutic toxicity.

Poor risk AML is a heterogeneous group of cytogenetic abnormalities with very limited treatment options and extremely low overall survival rates(1), even accounting for newer therapies (e.g. Venetoclax plus Azacitidine)(4, 5). While gene expression profiles – particularly those with single cell resolution – are improving our understanding of AML heterogeneity, the origins of relapse and revealing new clinical targets(31), the role of proteostasis has been comparatively understudied(44, 45). The selective reduction of leukemic cells by CKS1 inhibition demonstrates that precisely targeting proteostatic regulators can be a new frontier in AML therapy.

Here we demonstrate that CKS1 regulates LSC viability through RAC1/NADPH/ROS pathways, fundamental in amplifying extrinsic and intrinsic signals in normal hematopoiesis and AML(6, 46), and critical to metastatic disease across cancer(47). The balance of intracellular ROS in normal and malignant hematopoietic stem cells has been of great interest in recent years(37, 41), and changes in mitochondrial functions due to *RAS* mutations and nicotinamide-NAD metabolism underline the critical role for this pathway in primary patient resistance to Venetoclax(6, 7). The induction of ROS in AML upon CKS1 inhibition demonstrates that the balance of CKS1-dependent protein degradation is key to maintaining stress responses in AML. This, together with LSCs requiring low ROS to maintain their stem cell potential, explains the strong reduction in LSC frequency conferred by CKS1i in primary patient AML (Figures 2 and 7).

The effect of CKS1i on normal hematopoiesis is clearly different to the effects observed in AML (Figure 3). Indeed, cell cycle blockage is highly beneficial, as patients treated with induction chemotherapy, which targets cycling cells, suffer from severe toxicity and cytopenia upon treatment. Classical induction chemotherapy is known to reduce the pool of hematopoietic progenitors, whilst quiescent HSCs are refractory to treatment, but ultimately undergo senescence(48). It has previously been reported that deletion of *p27* in murine progenitors increased cycling and potency (49). In agreement, we found that increased p27 protein levels and the accompanying cell cycle arrest of HSPCs by CKS1i could prevent DA reduction of normal cells *in vivo* (Figures 3), and in the context of AML could rescue the reduction in HSCs induced by chemotherapy (Figure 7). Importantly, CKS1i treatment also induced changes in fundamental HSPC signalling pathways involved in stem cell potency and response to stress. The overall suppression of key growth and activation cellular markers lead to an opposite phenotype to that seen in AML cells, with a reduction in intracellular ROS and an increase in normal HSC frequency (Figure 4). In addition, CKS1i also rescues negative effects of induction chemotherapy on intestinal crypts (Supp. Fig. 6), a major issue associated with patient chemotherapeutic by-toxicity(34, 35). Considering that older poor risk AML patients (>65 years), who comprise the majority of AML cases, are ineligible for intensive chemotherapy (50, 51), the reduction in toxicity towards healthy tissue conferred by CKS1i during DA treatment has the potential to improve outcomes independent of direct AML effects.

The non-AML-intrinsic mechanism of action and effects on normal HSPCs by CKS1i may also implicate further components in the bone marrow niche. We and others have detailed the evolving bone marrow niche in hematological malignancies(52), and the diverse repertoire of proteostatic machinery affected by CKS1i has the potential to affect cell competition in the leukemic bone marrow microenvironment by affecting normal HSPCs as well as stromal components.

The main limitation of our study is that we focus on a poor risk AML cohort which, despite covering a variety of cytogenetic and FAB subtypes, does not cover the full heterogeneity of AML patients seen in the clinic. Further work will be needed to evaluate the efficacy of CKS1i on intermediate and good risk AML patient groups.

Thus, the inhibition of CKS1-dependent protein degradation holds excellent promise for AML therapy, both as a single agent towards *CKS1B^high^* AML, and in combination with induction chemotherapy in remaining AML cases.

Reports of *CKS1B* overexpression correlating with outcome in other solid cancer types(28, 30), and novel ways to modulate CKS1 activity(53), indicate that proteostatic targeting, through this axis, holds much hope for future cancer therapy.

## Methods

### Study design

This study aimed to investigate the sensitivity of poor risk AML – a sub-class of AML with few treatment options – to inhibition of CKS1-dependent protein degradation, as well as the potential side effect of this inhibitor on normal hematopoietic stem/progenitor cells. We have performed several experiments using different approaches to address these objectives. We first analyzed whether the effect of CKS1i correlates to the transcriptional level of *CKS1* in bulk AML samples. We also evaluated the level of CKS1 protein expression in leukemic stem cells using mass cytometry analysis. We then evaluated the effect of CKS1i on primary poor risk AML and on normal hematopoietic stem/progenitor cells *in vivo* using immunodeficient mice.

We also performed proteomic analysis on both normal and leukemic cells to investigate the mechanisms of action of CKS1i and used a RAC1 inhibitor (NSC23766) or N-Acetyl L Cysteine (NAC) to rescue the effects of CKS1i.

### Primary AML and UCB samples

AML samples were obtained after informed consent at St Bartholomew’s Hospital (London, U.K.) at the time of diagnosis as part of the Bart’s Cancer Institute Poor-Risk AML consortium. Full details of patient information are provided in Supplementary Table 1. Live mononuclear cells (MNCs) were isolated by density centrifugation using Ficoll-Paque (GE healthcare). Prior to culture or xenotransplantation, AML cells were depleted for T-cells using the Easysep T-cell depletion kit (StemCell Technologies). Umbilical Cord Blood (UCB) was obtained from full-term deliveries after informed consent, at the Royal London Hospital (London, U.K.). MNCs were isolated by density centrifugation using Ficoll-Paque (GE healthcare). Cells were selected for CD34^+^ using the Easysep CD34^+^ enrichment kit (StemCell Technologies). Purity was confirmed by flow cytometry. The collection and use of all human samples were approved by the East London Research Ethical Committee (REC:06/Q0604/110) and in accordance with the Declaration of Helsinki.

### Patient derived xenografts (PDX) and *in vivo* drug treatment

All animal experiments were performed under the project license (PPL 70/8904) approved by the Home Office of the UK and in accordance to the Francis Crick institute animal ethics committee and ARRIVE guidelines. NOD-SCID IL2Rynull (NSG) mice were originally a gift from Dr Leon Schultz (Jackson Laboratory Bar Harbor, Maine, USA). These mice were rederived and bred since then at The Francis Crick Institute Biological Resources Facility.

Primary AML samples (1x10^6^ – 5x10^6^ cells total) or UCB-CD34^+^ (5x10^4^ cells total) were injected intravenously (I.V.) into unconditioned 10-12 weeks old female or male NSG mice. After 4 weeks, engraftment was assessed by bone marrow aspiration from long bones whilst mice were under isoflurane anaesthesia. Mice were stratified according to engraftment and sex and assigned to treatment and control groups accordingly. Mice were treated as indicated with 10mg/kg CKS1i (Skp2-Cks1 E3 ligase inhibitor, Merck Millipore) intraperitoneal injection (I.P.) for 5 days, DA (doxorubicin/cytarabine, 1.5mg/kg/10mg/kg respectively, Sigma Aldrich), doxorubicin on days 1-3, cytarabine on days 1-5 co-injected I.V.(33). Mice were scored for engraftment over the experimental course by bone marrow aspiration and for overall survival according to U.K. home office license protocols and following CRUK guidance (>20% peak body weight loss, overt signs of sickness/mortality).

### Leukemic/Normal Long-term culture initiating cell (L-LTC-IC) assay

These experiments were performed as originally published by our group(54). For all co-culture experiments, MS-5 stromal cells were seeded two days prior to AML/UCB cell addition at 4x10^5^ cells/ml to reach confluence at the time of irradiation. One day prior to AML/UCB addition, MS-5 stromal cells were irradiated with 7Gy and culture media was exchanged. On the day of starting co-culture, AML cells were plated at 2x10^5^ cells/ml in meylocult H5100 (StemCell Technologies) supplemented with IL-3, G-CSF and TPO (all 20ng/ml; Peprotech). UCB cells were plated at 2x10^5^ cells/ml in myelocult H5100 (StemCell Technologies). Half media changes were performed once per week without disrupting the feeder layer. At the start of week two, indicated drug treatments were added at 2x concentration in the half media change once. For L-LTC-CAFC assays, all cells were harvested at day 14 and sorted for live hCD45^+^mSca-1^-^ cells. Resulting cells were seeded in co-culture with fresh MS-5 stromal cells in a 96 well plate in a limiting dilution range (200,000 to 1,000) in 10 replicates and cultured for a further 5 weeks. At the end of the co-culture period cobblestone area forming cells were scored and L-LTC-IC frequency was calculated using the ELDA (Extreme Limiting Dilution Analysis) function in the Statmod R package.

For LTC-IC assays, media was continuous changed each week until week five, when cultures were harvested and live hCD45^+^mSca-1^-^ cells were sorted. Resulting cells were seeded in co-culture with fresh MS-5 stromal cells in a 96 well plate in a limiting dilution range (10,000 to 100) in 10 replicates and cultured for a further three weeks. At week eight, myelocult H5100 was replaced with Methocult methycellulose (StemCell Technologies H4434) for a further two weeks, after which wells were scored for colony-forming units and LTC-IC frequency was calculated using the ELDA (Extreme Limiting Dilution Analysis) function in the Statmod R package.

### Protein translation assays

Protein translation was measured using the OP-Puromycin protein translation kit (Life Technologies). AML cell lines were seeded at 2x10^5^ cells/ml one day prior to treatment with the indicated drugs (day 0). The following day (day 1), drugs were added to culture wells at the indicated concentration. The next day (day 2), 10 μM OP-Puromycin was added to culture wells for one hour under culture conditions (37C, 5% CO_2_). Cells were washed three times in ice-cold PBS and fixed in 4% paraformaldehyde (Sigma Aldrich) at room temperature for 15 mins in the dark. Cells were washed three times in PBS and permeabilised in PBS + 0.5% Triton X-100 (Sigma Aldrich) for 15 mins. Cells were washed twice in Click-IT reaction buffer wash solution and stained as per the manufacturer’s instructions (Life Technologies). Abundance of OP-Puromycin was assessed using flow cytometry on a BD Fortessa FACS analyser.

### Intracellular ROS staining

Intracellular reactive oxygen species were assayed using the CellRox deep red reagent (Life Technologies). AML cell lines were seeded at 2x10^5^ cells/ml one day prior to treatment with the indicated drugs (day 0). The following day (day 1), drugs were added to culture wells. The next day (day 2), CellRox deep red was added to each well at a final concentration of 5uM and verapamil was added at a final concentration of 50 μM. Cells were continued to be incubated in the same conditions (37C, 5% CO_2_) for 1hr. After incubation, cells were collected from wells and washed three times in PBS + 1%FBS + 50 μM verapamil and finally resuspended in PBS + 1% FBS + 50 μM verapamil + DAPI (0.1μg/ml) before analysis on a BD Fortessa FACS analyser.

### NADP/NADPH assays

Total NADP/H and NADPH were measured using the NADP/NADPH colorimetric assay kit (Abcam). AML cell lines were seeded at 2x10^5^ cells/ml one day prior to treatment with the indicated drugs (day 0). The following day (day 1), drugs were added to culture wells at the indicated concentration and cells were harvested after 8 hours. All cells were collected from the wells and washed three times in ice-cold PBS. Cells were lysed in NADP/NADPH extraction buffer by performing two freeze/thaw cycles (20 mins on dry ice followed by 10 mins at room temperature). Lysates were centrifuged at 13,000g for 10minutes and the supernatant was retained. Lysate supernatant was split in half, with one half remaining on ice and the other half incubated at 60C for 30mins to remove NADP^+^. Total NADP/H (NADPt) and NADPH only lysates were run in 96 well plates with freshly made standards as per the manufacturers’ instructions. NADP/NADPH ratio was calculated as (NADPt-NADPH)/NADPH.

### Mass Cytometry

CyTOF preparation and analysis was carried out as per our previous publication (36). Cultured cells were washed in ice-cold PBS three times and incubated with 5μM Cisplatin (Fluidigm) to mark dead cells. Cells were washed three times in ice-cold PBS and fixed in 1.6% formaldehyde (Sigma Aldrich). Fixed cells were surface stained with the relevant antibodies (resources table) for two hours at room temperature followed by three washes with PBS. Cells were permeabilised in 1ml Perm buffer III (BD biosciences) on ice for 30mins, washed three times in ice-cold PBS and incubated with the relevant intracellular antibodies (resources table) overnight at 4 °C with gentle rotation. Resulting cells were wash three times in ice-cold PBS and stained with 100nM Iridium in PBS + 0.1% Saponin (Riedel-de Haen) overnight before analysis on a Helios Mass Cytometer (Fluidigm). All control and CKS1i treated samples were prepared simultaneously with equal buffers, antibodies and fixation.

### Publicly available datasets

*CKS1B* expression in normal and malignant hematopoiesis was obtained through Bloodspot.eu. Overall survival and stratification for *CKS1B* expression was calculated from data obtained from The Cancer Genome Atlas (TCGA). AML cell line RNA sequencing data was obtained from the EBI Expression Atlas (RNA-seq of 934 Human cancer cell lines from the Cancer Cell Line Encyclopedia).

### Statistics and data interpretation

Results shown are +/-SEM unless otherwise indicated. To compare treatment versus control in all *in vitro* and *in vivo* experiments, a Student’s *t-*test was used as indicated in the figure legend with N number indicated. For all comparisons, unpaired *t*-tests were undertaken unless otherwise indicated. All repeat samples presented are from biological replicates of distinct samples/xenotransplantations.

Survival analyses were carried out using the “survminer” package on R to calculate significance between Kaplan-Meier curves and Hazard ratios. Kaplan Meier graphs were plotted using Graphpad Prism.

Correlation analyses were carried out using the “performance analytics” and “corrplot” packages in R. Multiple DSS comparisons with *CKS1B* expression were carried out with pairwise complete observations using Spearman, Pearson and Kendall correlation coefficients. Individual correlations for *CKS1B* vs DSS or IC50 were plotted using Graphpad Prism.

Stem cell frequency was calculated using the extreme limiting dilution analysis (ELDA) function in the “statmod” R package(55).

Pathway analysis and enrichment was run through MetaCore (genego.com) and network interactions produced on String (string-db.org).

CyTOF analysis was conducted using the CATALYST package on gated live, single cells.

### Data Resources

The mass spectrometry proteomics data have been deposited to the ProteomeXchange Consortium via the PRIDE partner repository (PXD022754 and 10.6019/PXD022754).

## Supplementary Material

Supplementary Information

Supplementary Tables

## Summary

Targeting CKS1-dependent protein degradation has opposing effects in human normal and malignant hematopoiesis, protecting normal HSCs while reducing the leukemic stem cell pool.
