# Supplementary material for: CKS1 inhibition reveals vulnerabilities in leukemic stem cells with concomitant protection of healthy hematopoietic stem cells
Source: Sci Transl Med. Author manuscript; Available in PMC 2022 Jul 5. (PMC7612983; doi:10.1126/scitranslmed.abn3248)
Supplement: Supplementary Information [file EMS146397-supplement-Supplementary_Information.pdf]

Science Translational Medicine
AAAS

Supplementary Materials for

CKS1 inhibition depletes leukemic stem cells and protects healthy hematopoietic stem cells in acute myeloid leukemia

William Grey *et al.*

Corresponding author: Dominique Bonnet, dominique.bonnet@crick.ac.uk;
William Grey, william.grey@york.ac.uk

Sci. Transl. Med. **14**, eabn3248 (2022)
DOI: 10.1126/scitranslmed.abn3248

The PDF file includes:

Supplementary Methods
Figs. S1 to S13

Other Supplementary Material for this manuscript includes the following:

Tables S1 to S7
MDAR Reproducibility Checklist

Supplementary Information

Supplementary Methods

AML cell line, AML primary sample, UCB CD34$^+$ and MS-5 culture

All AML cell lines and MS-5 stromal cells were originally obtained from the ATCC and maintained by the Francis Crick Cell Services. Before using these lines, they were authenticated using the Short Tandem Repeat (SRF) profiling and tested for mycoplasma prior to commencing experiments. All AML cell lines were cultured in RPMI 1640, 10% heat-inactivated FBS and 1% penicillin/streptomycin (Life Technologies) at 37ºC, 5% CO_2. Umbilical cord blood CD34$^+$ cells were cultured in StemSpan SFEMMII (StemCell Technologies) supplemented with Human SCF (150ng/ml), Human FLT3 ligand (150ng/ml) and Human TPO (20ng/ml; all Peprotech) at 2x10^5 cells/ml at 37ºC, 5% CO_2. For relative viability, apoptosis and IC_{50} calculations cell lines were seeded in 96 well plates at a concentration of 2x10^5 cells/ml with the indicated dose of drug. Measurements of viability (% reduction O_2) or apoptosis (Annexin V positivity) were taken at 48 hours post-treatment. MS-5 stromal cells were cultured in IMDM, 10% heat-inactivated FBS and 2% penicillin/streptomycin (Life Technologies) at 37ºC, 5% CO_2. Primary human AML samples were recovered for 24 hours in StemSpan SFEMMII (Stem Cell Technologies) supplemented with IL-3, G-CSF, TPO (20ng/ml each; all Peprotech) and treated as indicated.

Mass Spectrometry

THP-1 AML cell lines and UCB CD34$^+$ cells were cultured as per culture and drug treatment in methods. Cells were recovered for 24 hours in their respective media followed by sub-lethal AML doses of CKS1i (1μM) for 12 hours. All cells were retrieved from wells, washed three times in ice-cold PBS and snap frozen in liquid nitrogen as dry pellets. Cells were cultured in the conditions above, with differing media compositions.

Cell pellets were lysed in 100 μL of urea buffer (8 M urea in 20 mM HEPES, pH: 8.0), lysates were further homogenized by sonication (30 cycles of 30s on 30s off; Diagenode Bioruptor Plus) and insoluble material was removed by centrifugation. Protein amount was quantified using BCA (Thermo Fisher Scientific). Then, 100 and 20 μg of protein for THP-1 and CD34$^+$ samples, respectively, were diluted in urea

buffer to a final volume of 300 μL and subjected to cysteine alkylation using sequential incubation with 10 mM dithiothreitol (DDT) and 16.6 mM iodoacetamide (IAM) for 1 h and 30 min, respectively, at 25 °C with agitation. Trypsin beads (50% slurry of TLCK-trypsin; Thermo-Fisher Scientific; Cat. #20230) were equilibrated with 3 washes with 20 mM HEPES (pH 8.0), the urea concentration in the protein suspensions was reduced to 2 M by the addition of 900 μL of 20 mM HEPES (pH 8.0), 100 μL of equilibrated trypsin beads were added and samples were incubated overnight at 37°C. Trypsin beads were removed by centrifugation (2000 xg at 5°C for 5 min) and the resulting peptide solutions were desalted using carbon C18 spin tips (Glygen; Cat. # TT2MC18). Briefly, spin tips were activated twice with 200 μL of Elution Solution (70% ACN, 0.1% TFA) and equilibrated twice with 200 μL of Wash Solution (1% ACN, 0.1% TFA). Samples were loaded and spin tips were washed twice with 200 μL of Wash Solution. Peptides were eluted into fresh tubes from the spin tips with 4 times with 50 μl of Elution Solution. In each of the desalting steps, spin tips were centrifuged at 1,500xg at 5C for 3 min. Finally, samples were dried in a SpeedVac and peptide pellets were stored at −80°C.

For mass spectrometry identification and quantification of proteins, samples were run twice in a LC-MS/MS platform. Briefly, peptide pellets were resuspended in 100 μL and 20 μL of reconstitution buffer (20 fmol/μL enolase in 3% ACN, 0.1% TFA) for THP-1 and $CD34^+$ samples, respectively. Then, 2 μL were loaded onto an LC-MS/MS system consisting of a Dionex UltiMate 3000 RSLC coupled to a Q Exactive Plus Orbitrap Mass Spectrometer (Thermo Fisher Scientific) through an EASY-Spray source (Cat. # ES081, Thermo Fisher Scientific). Mobile phases for the chromatographic separation of the peptides consisted in Solvent A (3% ACN: 0.1% FA) and Solvent B (99.9% ACN; 0.1% FA). Peptides were loaded in a micro-pre-column (Acclaim PepMap 100 C18 LC; Cat. # 160454, Thermo Fisher Scientific) and separated in an analytical column (Acclaim PepMap 100 C18 LC; Cat. # 164569, Thermo Fisher Scientific) using a gradient running from 3% to 23% over 120 min. The UPLC system delivered a flow of 2 μL/min (loading) and 300 nL/min (gradient elution). The Q-Exactive Plus operated a duty cycle of 2.1s. Thus, it acquired full scan survey spectra (m/z 375–1500) with a 70,000 FWHM resolution followed by data-dependent acquisition in which the 15 most intense ions were selected for HCD (higher energy collisional dissociation) and MS/MS scanning (200–2000 m/z) with a resolution of

17,500 FWHM. A dynamic exclusion period of 30s was enabled with a m/z window of ±10 ppms.

Peptide identification from MS data was automated using a Mascot Daemon 2.5.0 workflow in which Mascot Distiller v2.5.1.0 generated peak list files (MGFs) from RAW data and the Mascot search engine (v2.5) matched the MS/MS data stored in the MGF files to peptides using the SwissProt Database (SwissProt_2016Oct.fasta). Searches had a FDR of ~1% and allowed 2 trypsin missed cleavages, mass tolerance of ±10 ppm for the MS scans and ±25 mmu for the MS/MS scans, carbamidomethyl Cys as a fixed modification and PyroGlu on N-terminal Gln and oxidation of Met as variable modifications. Identified peptides were quantified using Pescal software in a label free procedure based on extracted ion chromatograms (XICs). Thus, the software constructed XICs for all the peptides identified across all samples with mass and retention time windows of ±7 ppm and ±2 min, respectively and calculated the area under the peak. Individual peptide intensity values in each sample were normalized to the sum of the intensity values of all the peptides quantified in that sample. Data points not quantified were given a peptide intensity value equal to the minimum intensity value quantified in the sample divided by 10. Protein intensity values were calculated by adding the individual normalized intensities of all the peptides comprised in a protein and values of 2 technical replicates per sample were averaged. Protein score values were expressed as the maximum Mascot protein score value obtained across samples.

Drug sensitivity and resistance testing (DSRT)

Single drug DSRT was performed as described previously*(52)*. In brief, compounds, each with 7 different concentrations, were pre-plated using an acoustic liquid handling Echo 550 (Labcyte) to 384-well plates. Primary AML cells were suspended in conditioned medium (RPMI 1640 supplemented with 10% fetal bovine serum, 2mM L-glutamine, penicillin-100U/ml, streptomucing-100ug/ml and 12.5% conditioned medium from HS-5 human bone marrow stromal cells), DNase I treated for 4h (Promega), filtered through a 70μm cell strainer (Thermo Fisher Scientific) to remove possible cell clumps, and viable cells were counted. Pre-plated compounds in each 384-well plate were dissolved in 5ul of conditioned medium using a MultiDrop Combi peristaltic dispenser (Thermo Fisher Scientific) and shaken for 5 minutes to dissolve

the compounds. AML cells were plated at 5,000 cells/well in 20ul, leading to a final volume of 25ul/well. Plates were gently shaken for 5 minutes to mix the cells with the compounds and incubated for 72 hours at 37ºC, 5% CO_2.
Cell viability was measured using the CellTiter-Glo assay (Promega) with a PHERAstar microplate reader (BMG-labtech). Data was normalised to negative (DMSO only) and positive control wells (100uM benzethonium chloride) and dose response curves calculated.
Ex vivo drug sensitivity of AML cells to the tested drugs was calculated using a drug sensitivity score (DSS), a modified form of the area under the inhibition curve calculation that integrates multiple dose response parameters for each of the tested drugs, as previously described*(53)*.

Intestinal crypt analyses

Tamoxifen (Sigma, #T5648) was dissolved in ethanol to 300 mg/ml and further diluted in sunflower seed oil (Sigma #S5007) to a final concentration of 30 mg/ml. To induce recombination, 6-14 weeks old Lgr5$^{tm1(cre/ERT2)Cle}$ mice were given one dose of tamoxifen (150 ug/g body weight) via oral gavage. After 24h, chemotherapy was administered as described above. After seven days the animals were culled, the intestines harvested and fixed in 10% neutral buffered formalin for 24h and subsequently transferred to 70% ethanol. After embedding and sectioning, the slides were stained with anti-EGFP (LGR5) or Ki67 and the number of positive crypts (LGR5) or cells per crypt (Ki67) were counted.

AML cell line in vivo experimentation

AML cell lines were transduced with GFP-Luciferase containing vectors as per our previous reports *(41)*. For both cell lines (THP-1 and HL60) $2x10^6$ cells were injected I.V. into unconditioned 10-12 weeks old female or male NSG mice. After 7 days engraftment was assessed by bioluminescence imaging. Isofluorane anesthetized mice were imaged 5-10 minutes post D-luciferin injection I.P. (15mg.kg; Caliper life sciences) using the Xenogen IVIS imaging system. Photons emitted were expressed as Flux (photons/s/cm^2), and quantified and analysed using “living image” software (Caliper life sciences).

Colony forming units

For resident mouse hematopoietic cell response to 5-FU', CKS1i, DA and DAC, colony forming ability was assessed in methylcellulose (StemCell Technologies M3434-GF). 10^4 $mCD45^+$ cells were sorted from PDX mice at the indicated points and seeded in methylcellulose and scored to colony forming units after 7 days. Cultures were dissolved in PBS, counted and 10^4 cells were re-seeded for passage 2 and passage 3.

Viability assays

Relative cell viability was assessed by % reduction O_2 in culture wells using the Alamar blue cell viability reagent (Life Technologies). Cells were seeded in 96 well plates at $2x10^5$ cells/ml and the indicated dose of drugs were added on top and incubated for 48 hours. Alamar blue reagent was added on top of cells, and cells were incubated for another 4 hours under the same conditions (37ºC, 5% CO_2). Plates were read on a spectramax plate reader (Biostars) at 570nm and 600nm and % reduction O_2 was calculated as per the manufacturer's instructions.

Flow Cytometry, apoptosis and cell cycle assays

Flow cytometry analysis was performed using a BD Fortessa flow cytometer (BD biosciences). Cells were prepared by washing in PBS + 1% FBS three times before staining in the same media with the indicated cell surface antibodies (resources table) for 1 hour at 4C. For apoptosis assays, cells were incubated with annexin V binding buffer in addition to the washing media (BD biosciences), washed three times in PBS + 1% FBS + 1x annexin V binding buffer and incubated with 0.1μg/ml DAPI prior to flow cytometry analysis. For cell cycle analysis, cells were washed three times in PBS + 1% FBS and fixed in BD fix/perm buffer (BD biosciences) for 20 minutes at room temperature. Cells were washed three times in BD perm/wash buffer + 0.1% Triton X-100 (BD biosciences) and incubated with intracellular antibodies, such as anti-Ki67, for 4 hours at 4C. Cells were washed three times in BD perm/wash buffer and 0.5 μg/ml DAPI was added for 15 minutes prior to analysis. For all flow cytometry, cells were initially identified based on forward and side scatter.

RNA extraction, reverse transcription and real time quantitative PCR (RT-qPCR)

Total RNA was isolated from patient samples after thawing, density centrifugation and T-cell depletion, using a RNeasy mini kit (Qiagen). Resulting RNA was reverse transcribed to produce cDNA using the Superscript III reverse transcriptase kit (Thermo Fisher Scientific) with oligoDT$_{20}$ primers (Sigma Aldrich). RT-qPCR experiments were performed with an ABI-7500 FAST Thermal Cycler (Applied Biosystems) using SYBR Green (Thermo Fisher Scientific). RNA abundance was quantified by the Comparative CT method with two independent control genes (*GAPDH* and *B-ACTIN*, *GAPDH* presented). The CT values used for each patient sample were the result of three technical triplicates. Primers are described in the resources table.

RAC1/RHOA G-LISA assay

Analysis of RAC1/RHOA-GTP abundance was carried out using the RAC1/RHOA G-LISA assay as per the manufacturer's instructions (Cytoskeleton inc.). Control and CKS1i treated AML cells were lysed on ice with the provided lysis buffer for 10 minutes and centrifuged at 10,000g, 4°C, for 5 minutes. Protein was quantified and normalized with precision red protein reagent. Lysate, lysis buffer only or control protein was incubated with G-LISA wells at 4°C for 30 minutes with agitation. Wells were washed three times with wash buffer and primary antibody incubation was carried out at room temperature for 45 minutes with agitation. Wells were washed three times with wash buffer and secondary antibody incubation was carried out at room temperature for a further 45 minutes with agitation. HRP detection reagent was added to each well and incubated at room temperature for 20 minutes (RAC1) or 15 minutes at 37C (RHOA) in the dark followed by measurement at 490nm.

Supplementary Figures

Supplementary Figure 1.

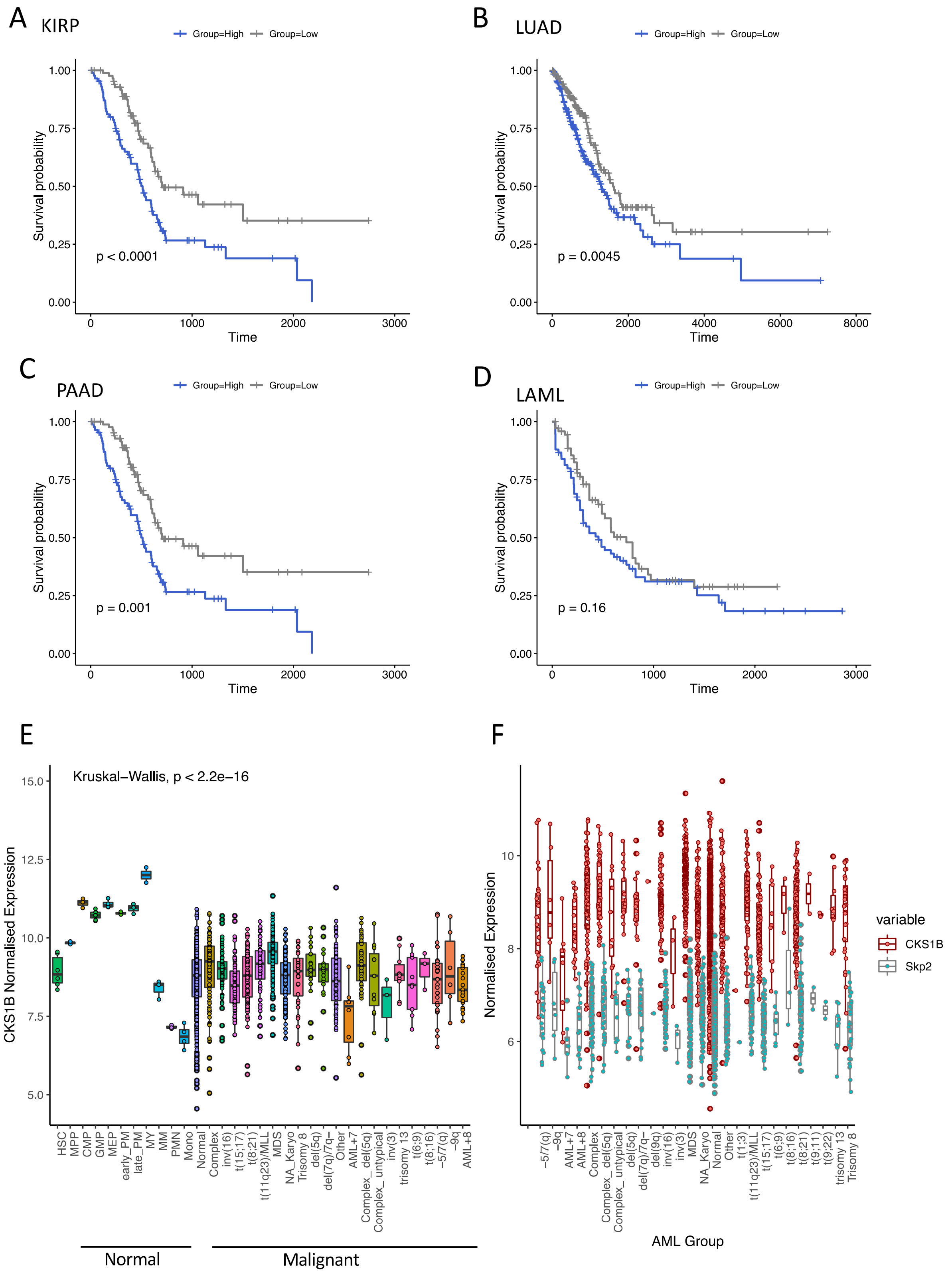

Supplementary Figure 1. Expression of *CKS1B* across publicly available datasets. A-D. Overall survival of TCGA patients stratified for *CKS1B* expression (50th percentile). Cohorts are as follows: KIRP = Kidney Renal Papillary Cell Carcinoma, LUAD = Lung Adenocarcinoma, PAAD = Pancreatic Adenocarcinoma, LAML = Acute Myeloid Leukemia. **E** *CKS1B* normalized expression and **F.** *SKP2* compared to *CKS1B* normalized expression of normal and malignant hematopoeitic cells obtained from Bloodspot.eu. Data sources: Human normal hematopoiesis (GSE42519), Human AML (GSE13159, GSE15434, GSE61804, GSE14468 and The Cancer Genome Atlas; TCGA).

Supplementary Figure 2.

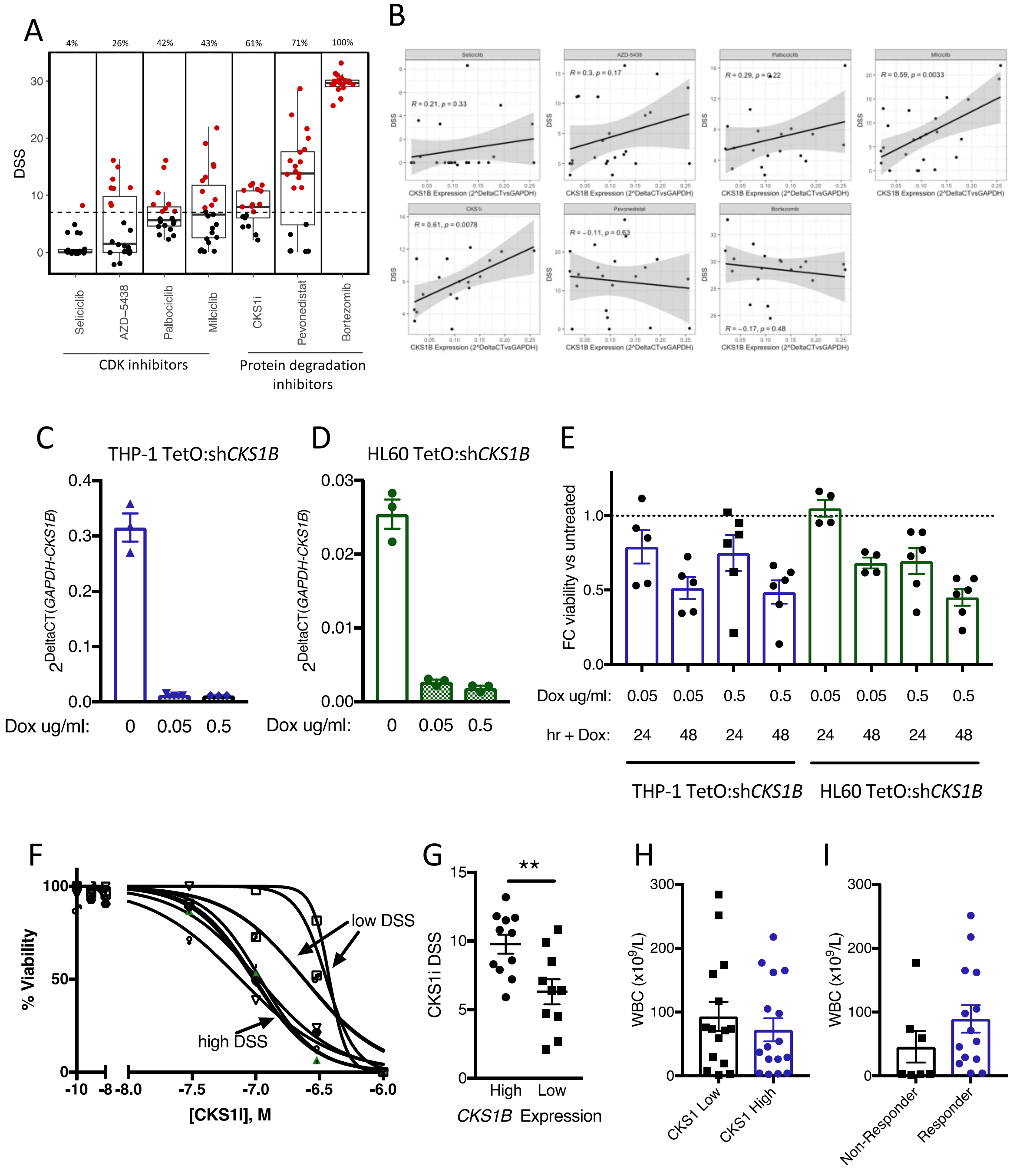

Supplementary Figure 2. Analysis of drug and genetic targeting of CKS1 in primary AML samples and AML cell lines A. Drug sensitivity score (DSS) for CDK

and protein degradation inhibitors in primary AML samples. Red indicates robust DSS (>7), percentage above indicates proportion of patients with robust response. **B.** Correlation between patient AML CKS1i drug sensitivity (DSS) and *CKS1B* expression for the indicated drugs. 95% confidence intervals presented. Pearson's correlation coefficient was calculated for correlation (R^2) and significance (P). Expression of *CKS1B* in **C.** THP-1 and **D.** HL60 cells transduced with TetO:shRNA:*CKS1B* in response to the indicated doses of doxycyclin after 24 hours. **E.** Fold change viability compared to uninduced control THP-1 (Blue) and HL60 (Green) cells transduced with TetO:shRNA:*CKS1B* in response to the indicated doses of doxycyclin for the indicated time points. **F.** Example dose dependent response curves for primary patient AML samples, indicating patient samples with high and low. **G.** CKS1i DSS grouped by *CKS1B* expression cut at the 50th percentile. White blood cell counts (x10^6/L) of patients with AML comparing **H.** *CKS1B* high versus low expression and **I.** CKS1i responders versus non-responders. A Student's *t*-test was used to calculate significance of difference for all graphs unless otherwise stated. ** P<0.005.

Supplementary Figure 3.

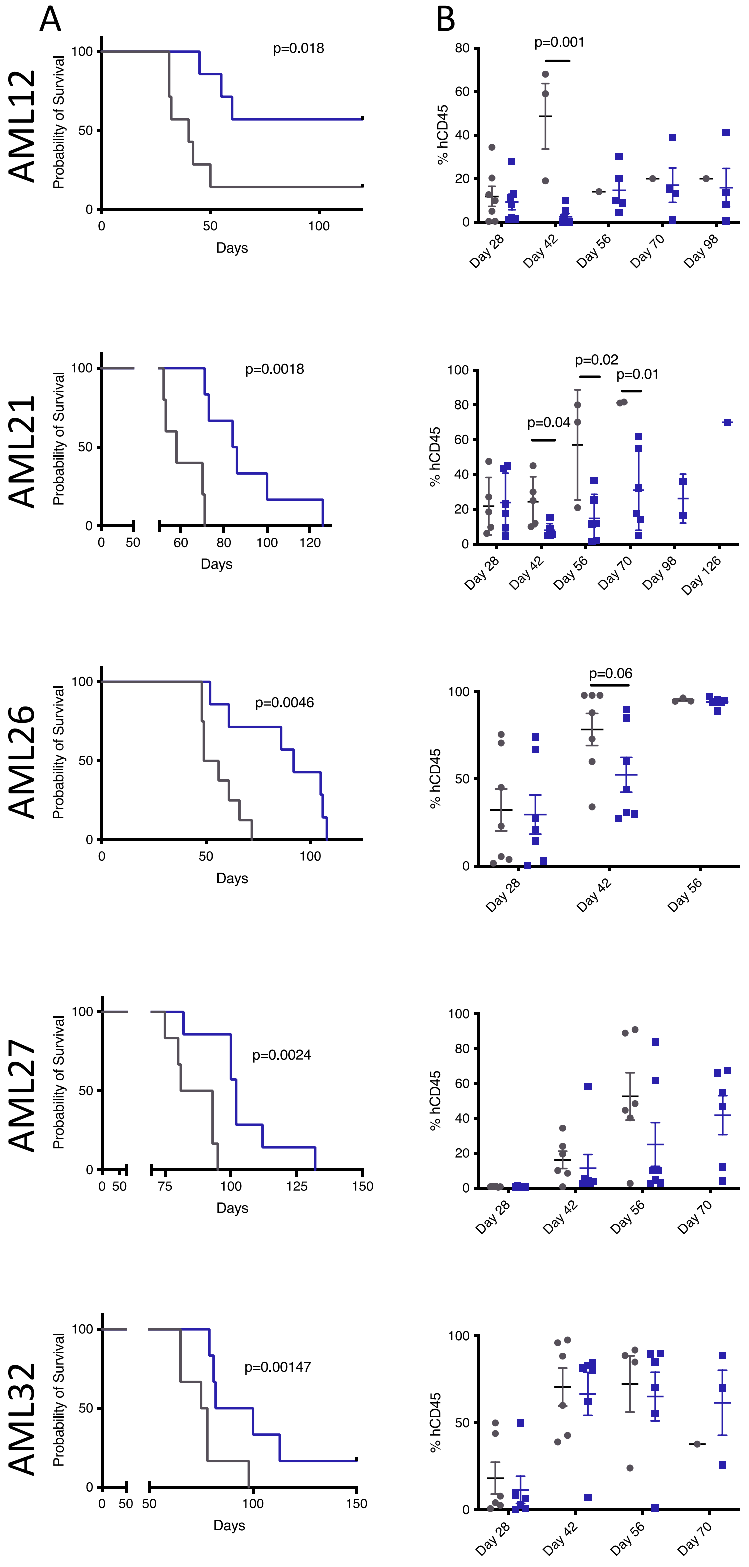

Supplementary Figure 3. Overall survival and bone marrow engraftment of patient derived xenografts. A. Kaplan Meier plots representing overall survival and **B.** Serial bone marrow aspirations for primary patient AML engrafted in NSG mice (Control = Grey, CKS1i treated = Blue, AML12 Control n = 7 CKS1i n = 7, AML21 Control n = 5 CKS1i n = 6, AML26 Control n = 7 CKS1i n = 7, AML27 Control n = 6 CKS1i n = 7, AML32 Control n = 6 CKS1i n = 6).

Supplementary Figure 4.

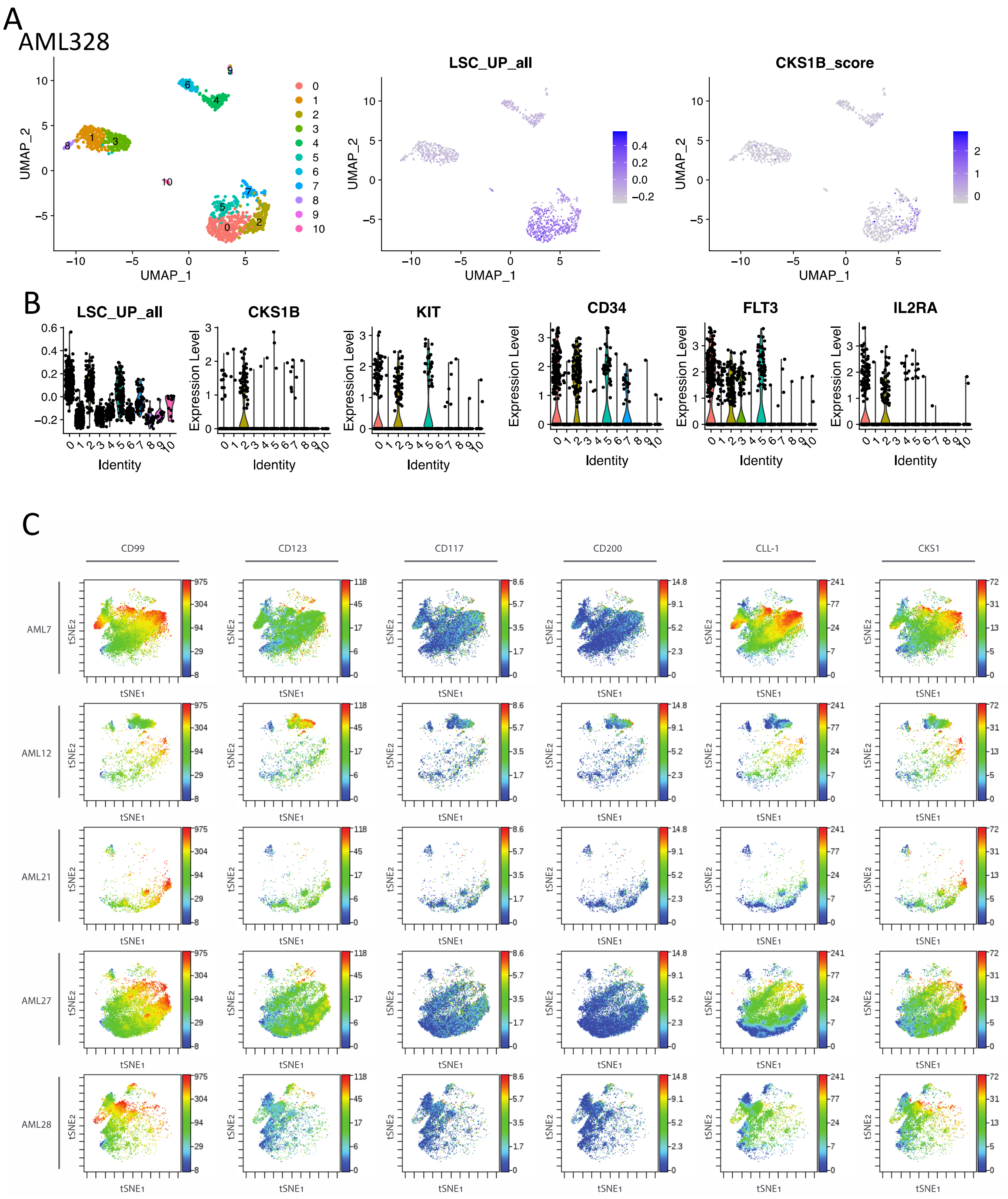

Supplementary Figure 4. Analysis of CKS1 expression in AML LSCs. A-B. Single cell RNAseq analysis for patient AML328 obtained from van Galen *et al.* (2019). Analyses present UMAP reductionality for cluster assignment, aggregated expression of “LSC up” gene score from Ng *et al.* (2016), *CKS1B* expression and violin plots for “LSC up” and individual genes. **C.** *t*-stochastic neighbour embedding of the indicated patients from CyTOF analyses. All markers were used for dimensionality reduction, key LSC cell surface markers and CKS1 are presented.

Supplementary Figure 5.

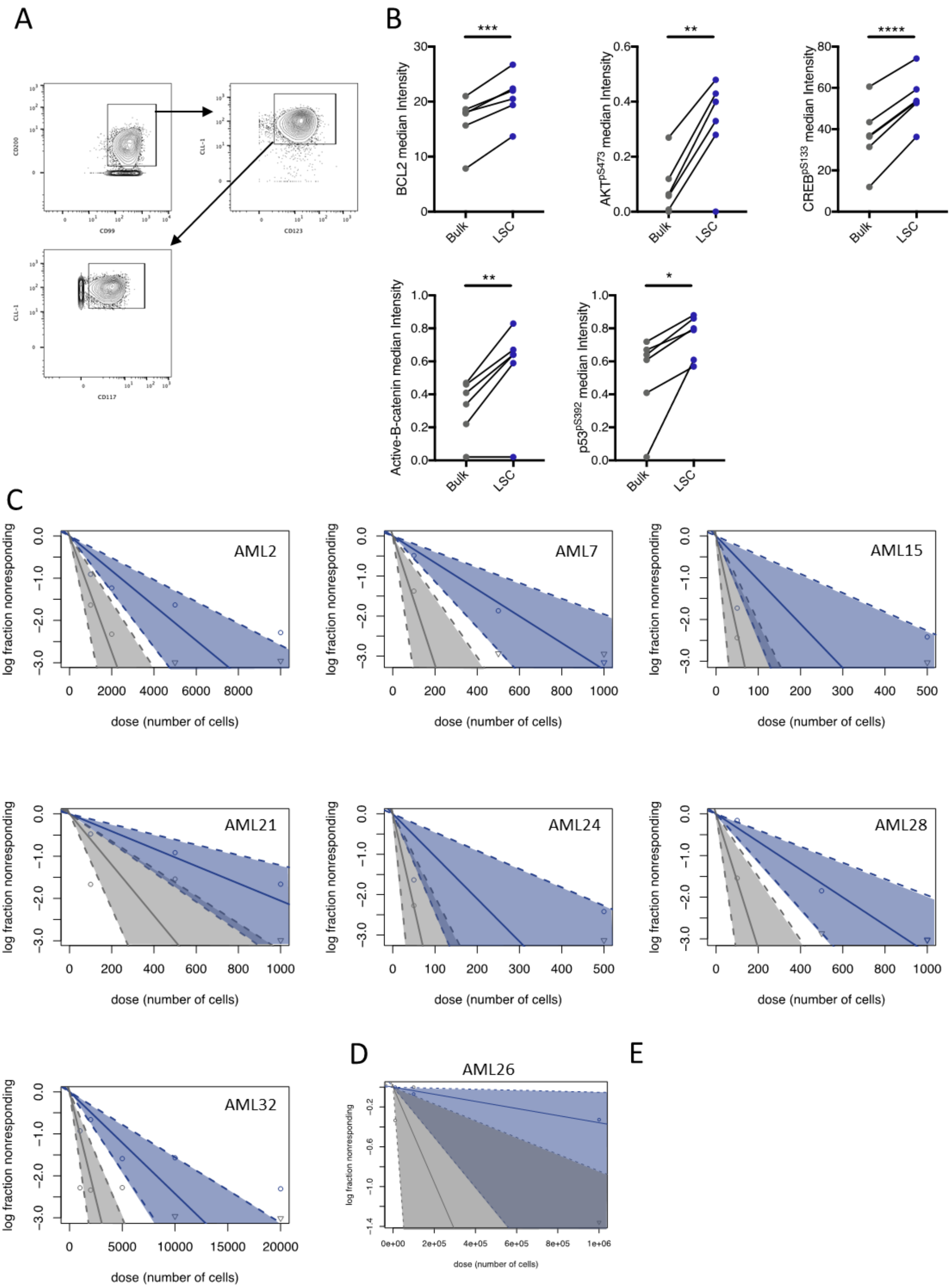

Supplementary Figure 5. Patient AML LSC response to CKS1i. A. Gating strategy for defining LSCs in bulk AML samples. Cells were gated for live, single cells and de-barcoded before example gating. **B.** Median intensity of the indicated proteins from CyTOF analyses of Bulk AML and LSCs. **C.** Graph of estimated L-LTC-IC frequency for the indicated patients control (grey) and treated with CKS1i (blue). **D.** Graph of estimated LSC frequency for AML patient 26 treated in the primary xenograft with control (grey) or CKS1i (blue). **E.** Cell cycle profiles of the indicated AML cell lines in response to CKS1i after 24 hours.

Supplementary Figure 6

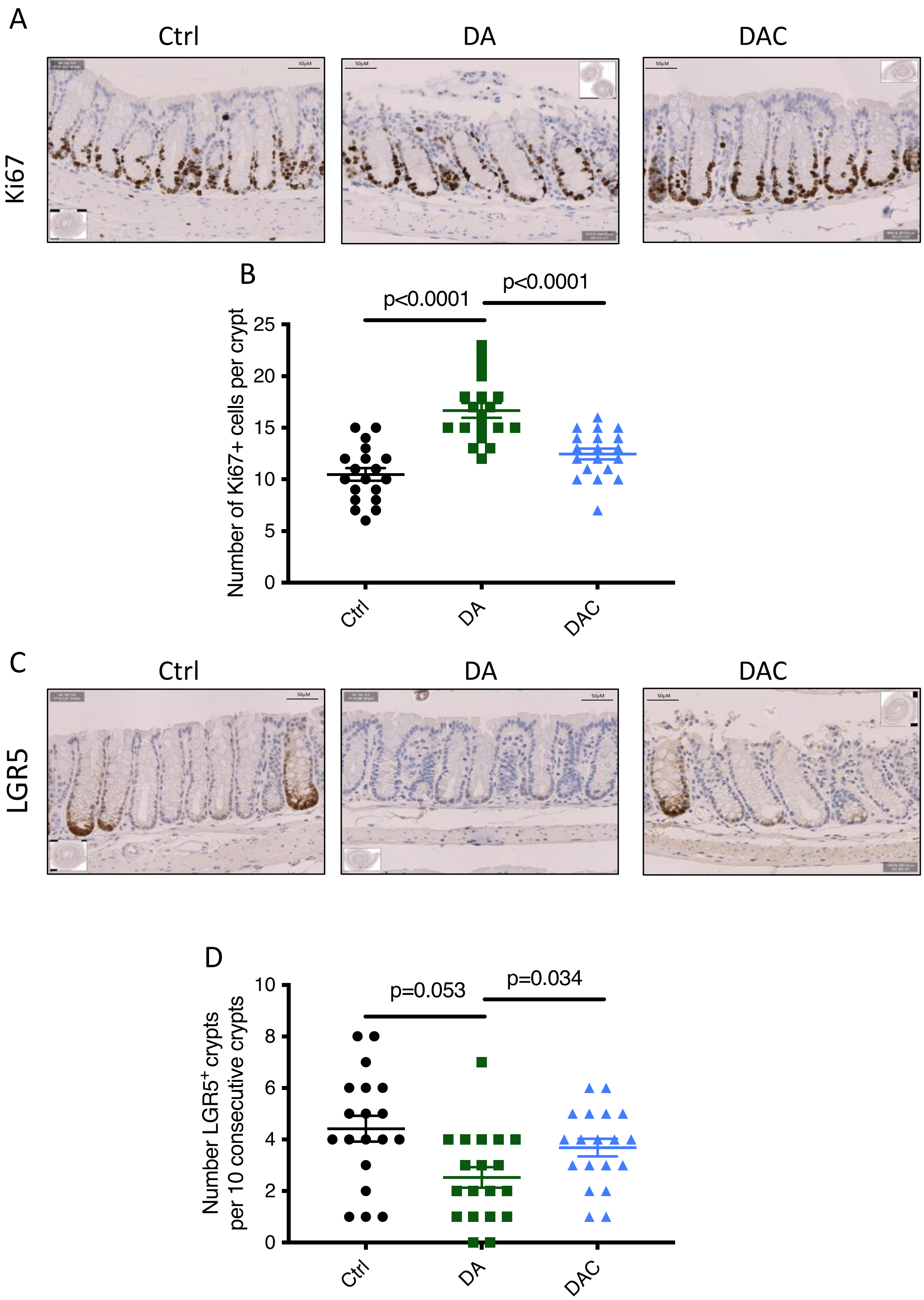

Supplementary Figure 6 Effect of combination chemotherapy on mouse intestinal crypts. A. Representative intestinal crypts stained with Ki67 and **B.** Number of Ki67 positive cells per crypt for the indicated treatments. **C.** Representative intestinal crypts stained for anti-GFP in LGR5-GFP mice and **D.** Number of LGR5 positive crypts per 10 consecutive crypts in intestinal preparations.

Supplementary Figure 7

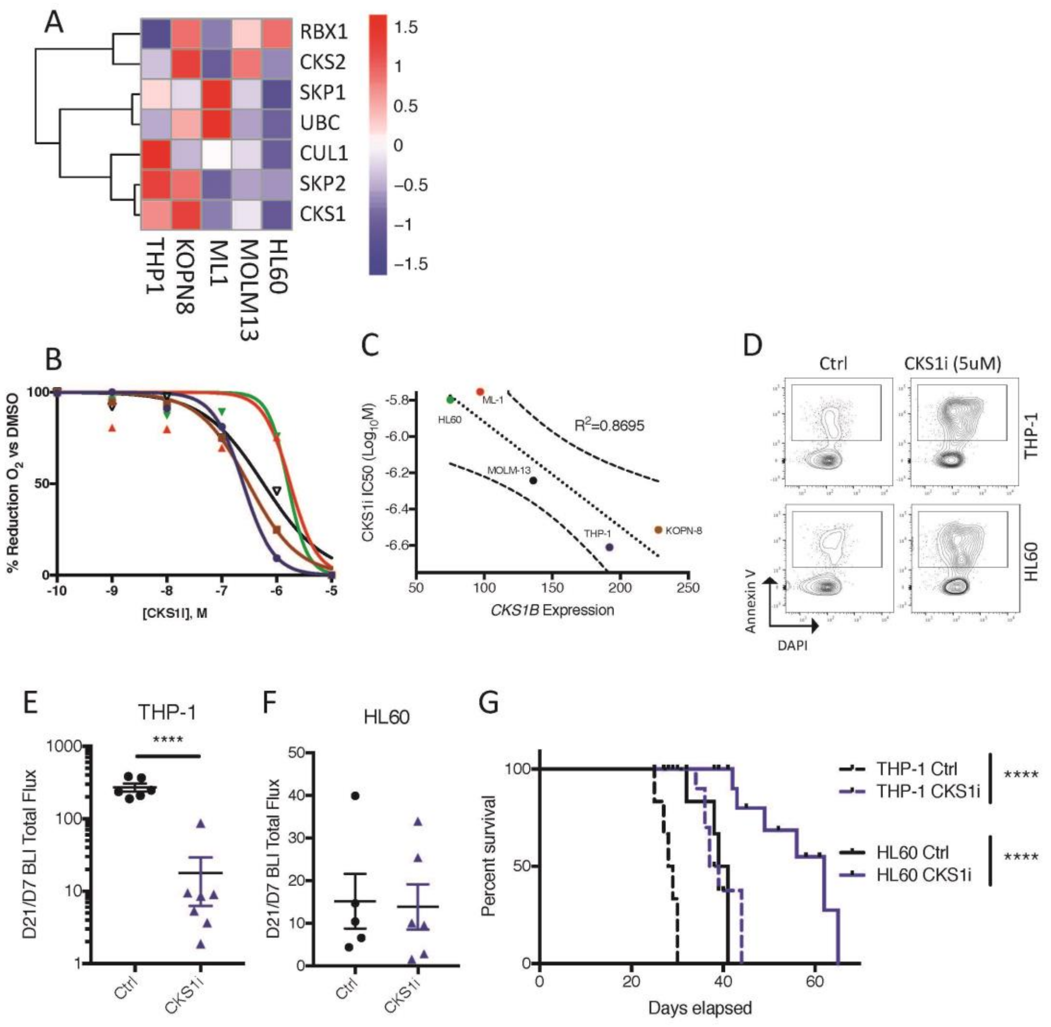

Supplementary Figure 7. AML cell line *CKS1B* expression dictates CKS1i sensitivity. A. Expression of key $SCF^{SKP2\text{-}CKS1}$ subunits in leukemic cell lines used in this study. Data presented are *z*-normalised (per gene) transcripts per million reads (TPMs) from the EBI Cell Line Expression Atlas. **B.** Percentage viability of AML cell lines cultured for 48 hours with indicated doses of CKS1i (*n*=3 for all cell lines on graph). **C.** Correlation between AML cell line CKS1i IC_{50} and *CKS1B* expression. 95% confidence intervals presented. Pearson's correlation coefficient was calculated for correlation (R^2). **D.** Representative FACS plots for induction of apoptosis in the indicated AML cell lines by presence of annexin V at the cell surface in response to CKS1i (5μM) at 48 hours. Fold change in vivo leukemic burden of **E.** THP-1 (Ctrl *n*=6, CKS1i *n*=7) and **F.** HL60 (Ctrl *n*=5, CKS1i *n*=6) cells day 21 (9 days post-CKS1i) versus day 7 (pre-CKS1i) expressed as bioluminescent total flux intensity. **G.** Overall

survival of xenografts carrying THP-1 and HL60 cell lines control or treated with CKS1i. A Student's *t*-test was used to calculate significance of difference for all graphs unless otherwise stated. **** $P<0.00005$.

Supplementary Figure 8

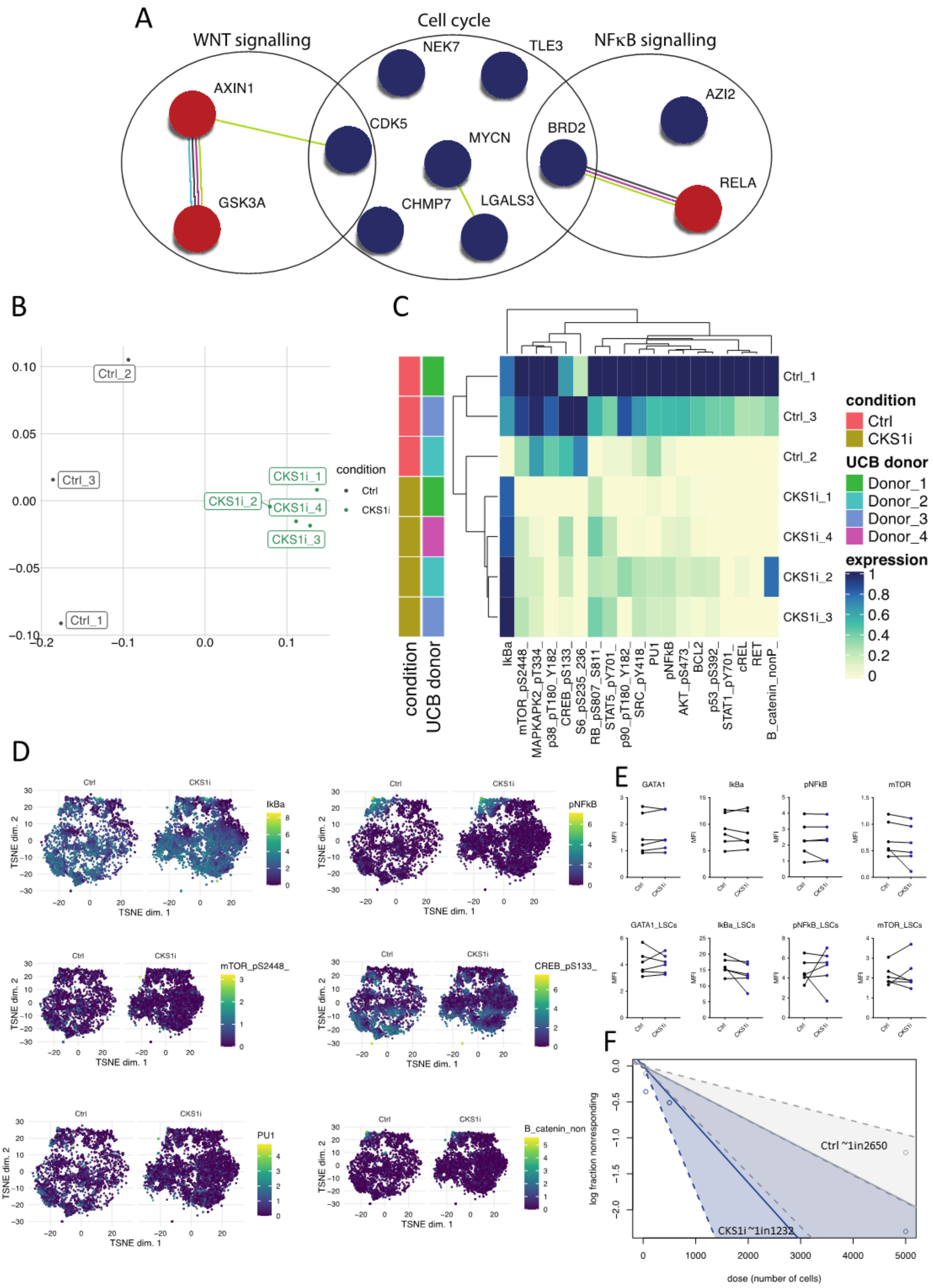

Supplementary Figure 8. Effect of CKS1i on healthy hematopoiesis. A. Key proteins differentially abundant in $CD34^+$ cells in response to CKS1i (Red = upregulated, Blue = downregulated). **B.** Pseudo-bulk-level multidimensional scaling (MDS) plot for all markers used in mass cytometry analyses. **C.** Unsupervised heatmap representing intracellular signalling markers in mass cytometric analyses *z*-scaled for each marker. **D.** *t*-distributed stochastic neighbor embedding for control vs CKS1i CyTOF samples with intensity scale for the indicated intracellular markers. **E.** Intracellular signalling components measured in primary AML bulk (top panel) or LSCs (bottom panel) post CKS1i treatment (1μM). **F.** LTC-IC estimated frequency of $CD34^+$ cells control (Grey) or treated with CKS1i (1μM, Blue).

Supplementary Figure 9

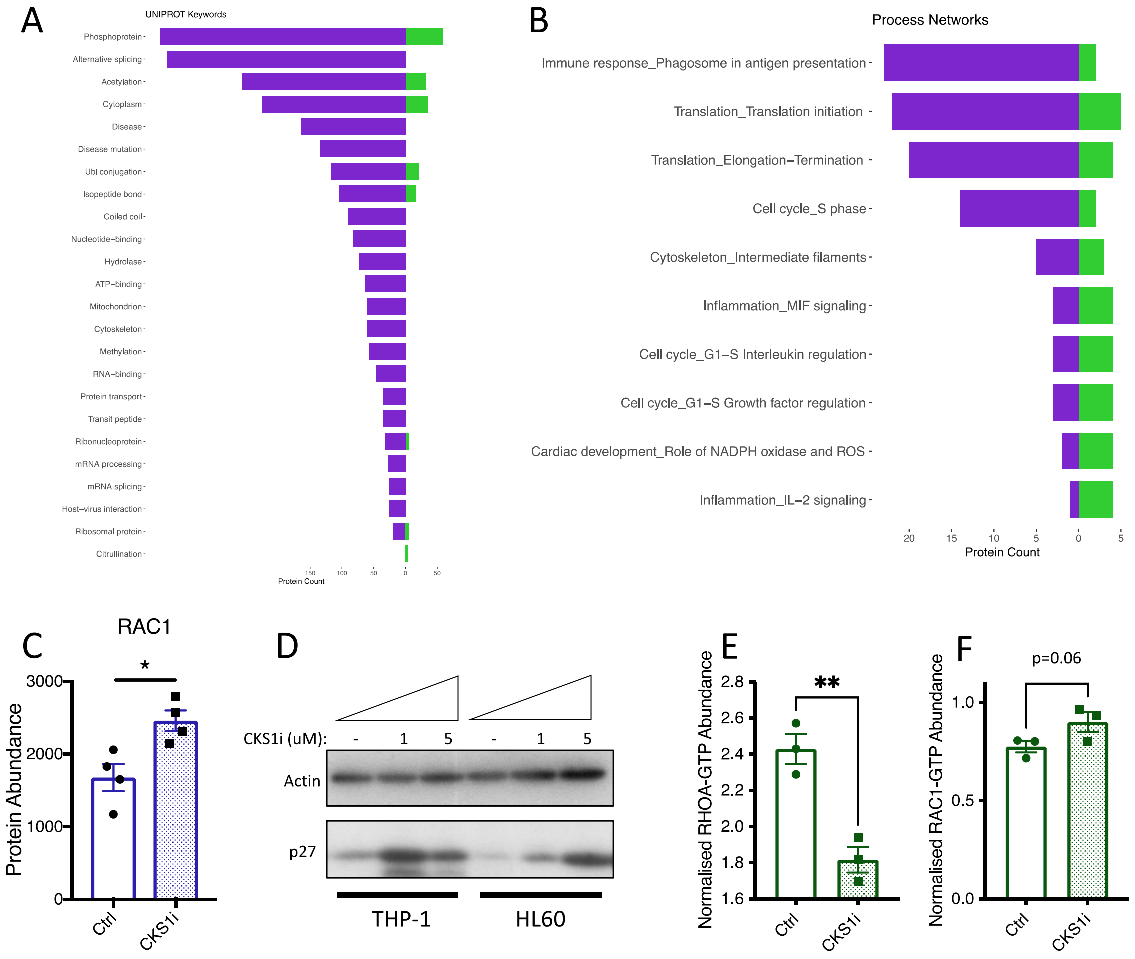

Supplementary Figure 9. Effect of CKS1i on AML cell lines. A. Uniprot keywords and **B.** Process networks from differentially abundant proteins in THP-1 (purple) and $CD34^+$ (green) cells. **C.** Abundance of RAC1 protein in THP-1 cells treated with CKS1i (1μM) from mass spectrometry analyses. **D.** Western blot for p27 in AML cell lines in response to the indicated doses of CKS1i after 24 hours. **E.** RHOA-GTP and **F.** RAC1-GTP abundance in HL60 cells treated with CKS1i (1μM). A Student's t-test was used to calculate significance of differences. * P<0.05, ** P<0.005.

Supplementary Figure 10

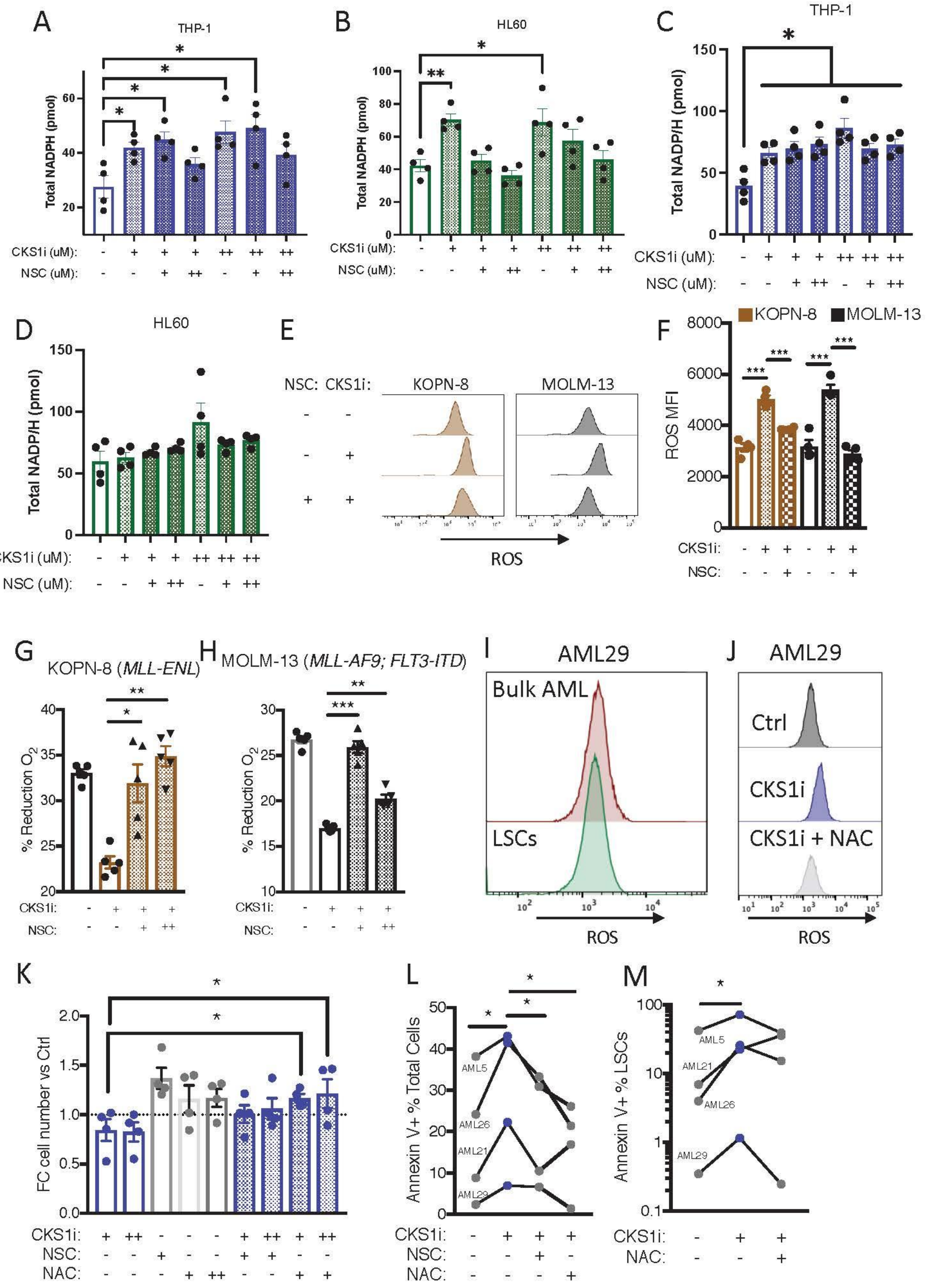

Supplementary Figure 10. CKS1i induces NADPH accumulation and lethal ROS in AML. Total NADPH (pmol) in **A.** THP-1 and **B.** HL60 cells treated for 8 hours with the indicated doses of CKS1i (+ = 1μM, ++ = 5μM) and NSC (+ = 0.1μM, ++ = 1μM). Total NADP/NADPH (pmol) in **C.** THP-1 and **D.** HL60 cells treated for 8 hours with the indicated doses of CKS1i (+ = 1μM, ++ = 5μM) and NSC (+ = 0.1μM, ++ = 1μM). **E.** Representative flow plots and **F.** Quantified mean fluorescence intensity of intracellular reactive oxygen species (ROS) in the indicated cell lines in response to CKS1i (+ = 1μM) and NSC (+ = 0.1μM) treatment (*n*=3 per cell line and treatment). Viability represented by percentage reduction O_2 of **G.** KOPN-8 and **H.** MOLM-13 cells in response to the indicated concentrations of CKS1i and NSC (*n* =5 per cell line and treatment, except THP-1 where *n* =6), CKS1i (+ = 1μM) and NSC (+ = 0.1μM, ++ = 1μM). **I.** Intracellular ROS measured in primary patient AML bulk vs LSC fraction. **J.** Intracellular ROS measured in primary AML cultured in control conditions, with CKS1i (1μM) or CKS1i + NAC (1μM + 1.25mM). **K.** Fold change absolute live cell number of patient AMLs compared to controls for the indicated treatments (CKS1i + = 1μM, ++ = 5μM, NSC + = 0.1μM, NAC + = 1.25mM, ++ = 2.5mM). Each point represents one primary patient AML sample. Percentage of annexin V positive cells of **L.** total primary patient AMLs and **M.** immunophenotypic LSCs with the indicated treatments (CKS1i + = 1μM, ++ = 5μM, NSC + = 0.1μM, NAC + = 1.25mM, ++ = 2.5mM). A Student's *t*-test was used to calculate significance of difference for all graphs * $P<0.05$; ** $P<0.05$; *** $P<0.005$.

Supplementary Figure 11

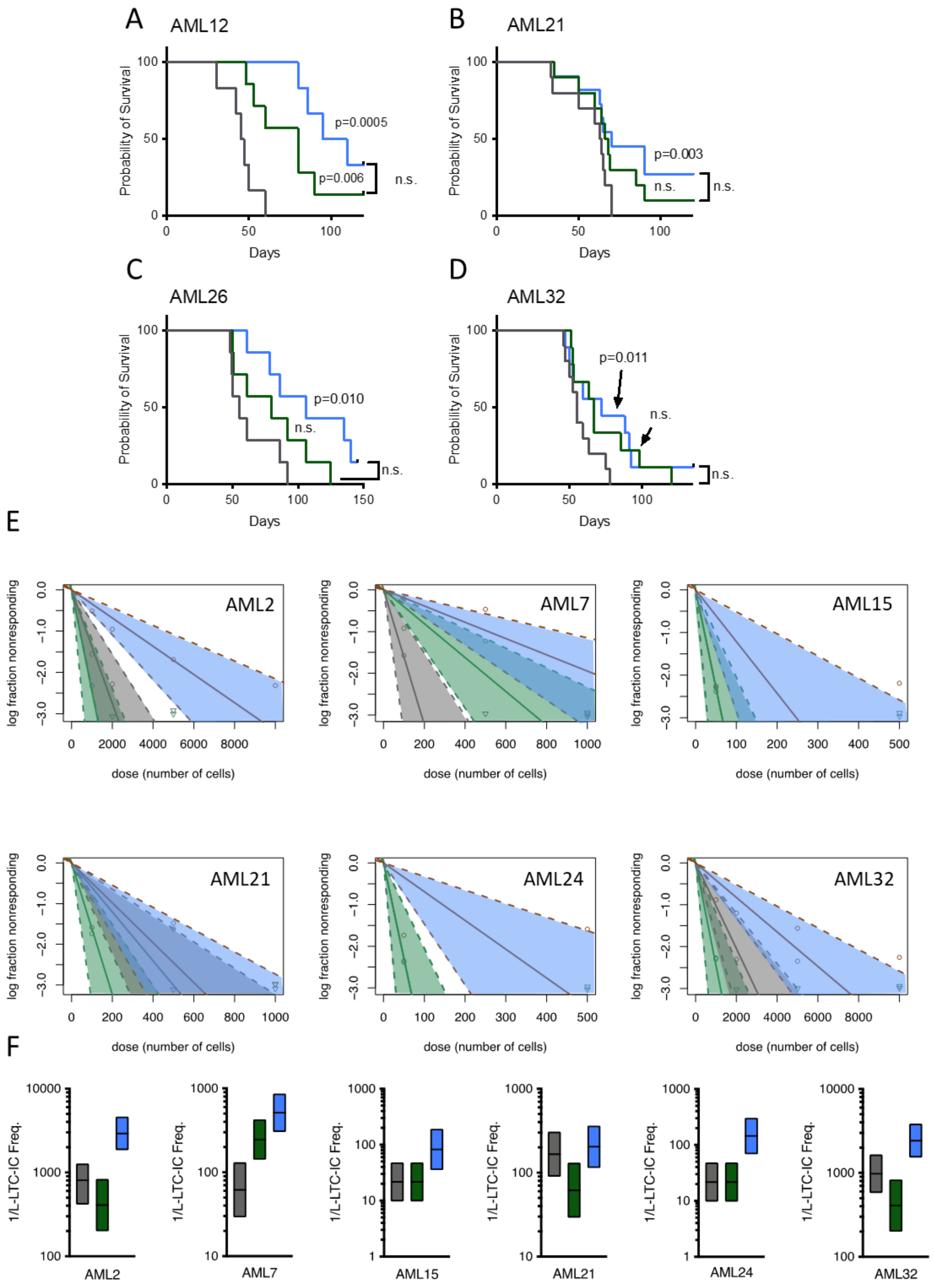

Supplementary Figure 11. In vivo and ex vivo response of patient AML samples to CKS1i. A-D. Kaplan Meier graphs for the indicated patient AML xenograft cohorts (Grey = control, green = DA, blue = DAC, AML12 control *n* = 6 DA *n* = 7 DAC *n* = 7, AML21 control *n* = 10 DA *n* = 10 DAC *n* = 11, AML26 control *n* = 7 DA *n* = 7 DAC *n* = 7, AML32 control *n* = 10 DA *n* = 9 DAC *n* = 9). **E.** Graph of estimated L-LTC-IC frequency for the indicated patients' control (Grey) and treated with DA (Green) or DAC (Blue). **F.** Calculated L-LTC-IC frequencies and confidence intervals by ELDA (Control = Grey, DA = Green, DAC = Blue).

Supplementary Figure 12

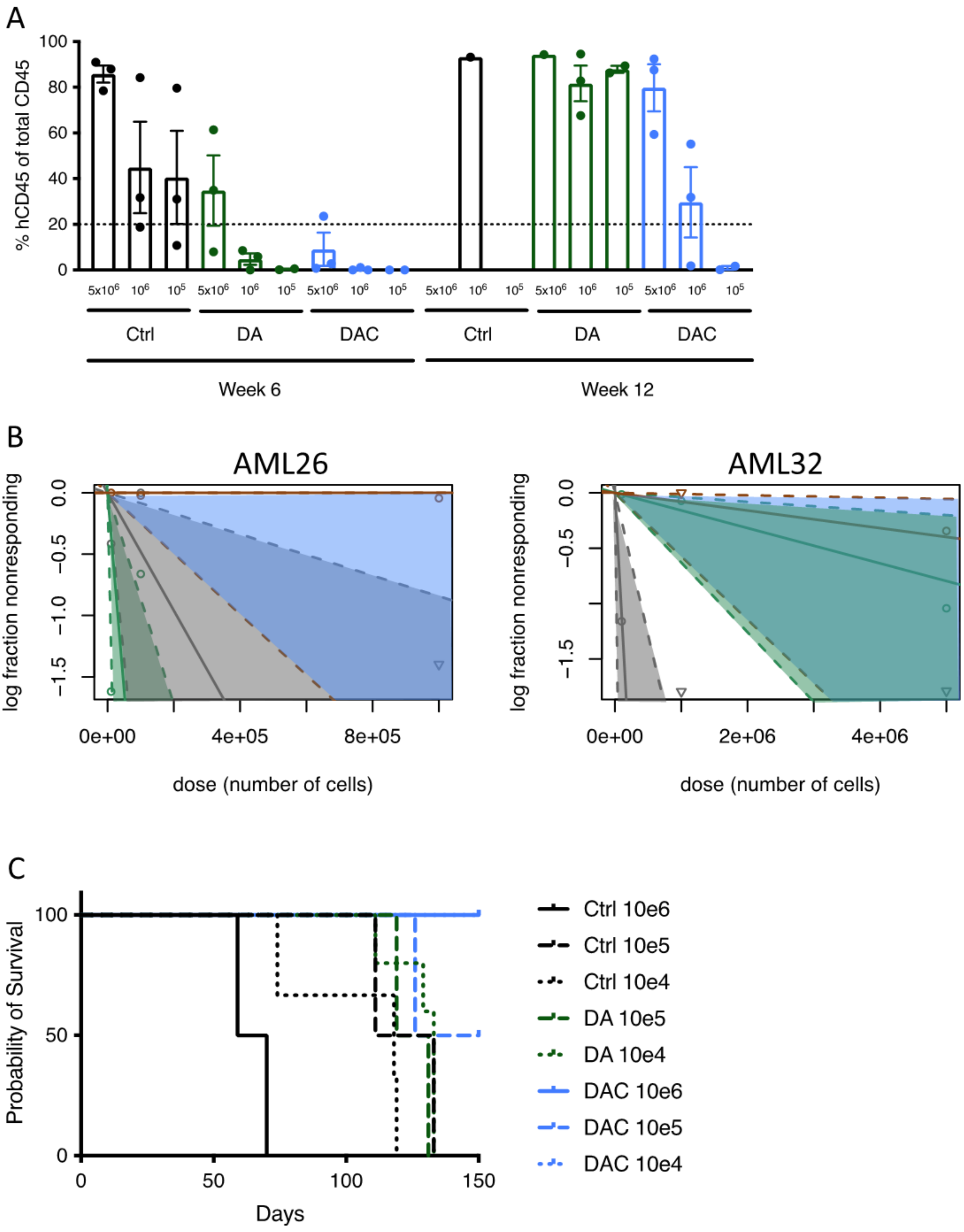

Supplementary Figure 12. Secondary transplantation of patient AML samples previously treated with chemotherapy. A. Percentage hCD45 bone marrow engraftment of AML32 engrafted in secondary mice at limiting dilution weeks 6 and 12 (Ctrl *n* = 3 per dose, DA $5x10^6$ & 10^6 *n* = 3 per dose; 10^5 *n* = 2, DAC $5x10^6$ & 10^6 & 10^5 *n* = 3 per dose). **B.** Graph of estimated LSC frequency for the indicated patients' control (Grey) and treated with DA (Green) or DAC (Blue). **C.** Overall survival of secondary transplantation mice from primary AML32 PDX control (Black) or treated with DA (Green) or DAC (Blue).

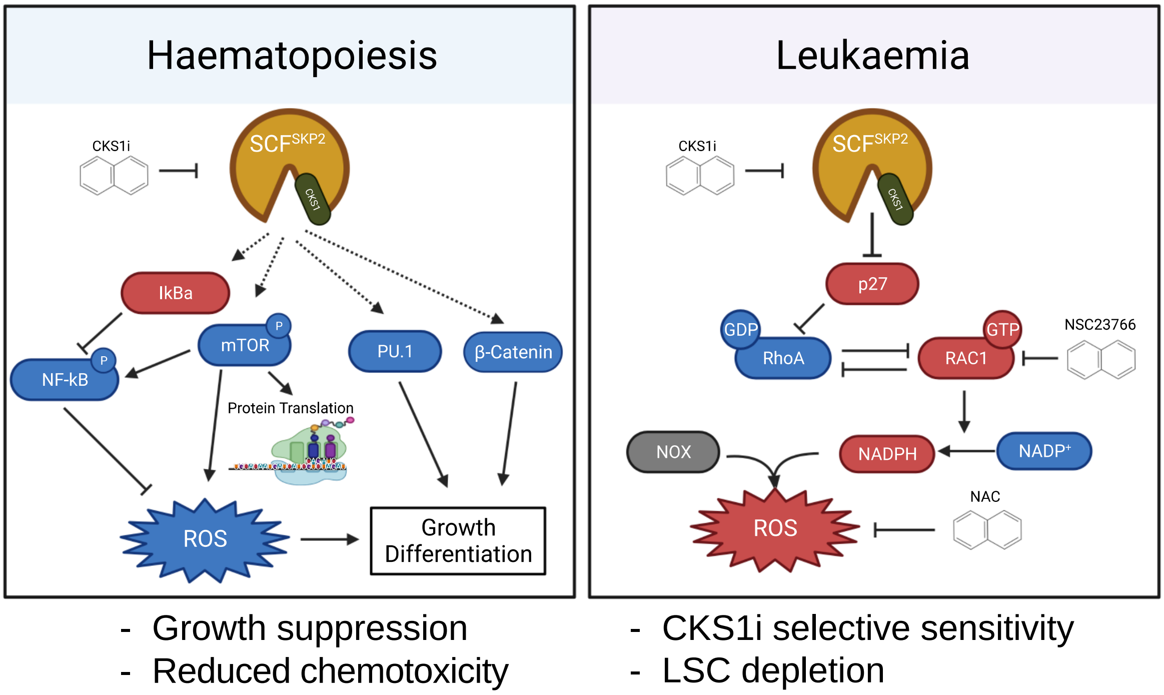

Supplementary Figure 13. Graphical abstract. Model for mechanism of action for CKS1i in healthy hematopoiesis and leukemia.
